# Family-based transcriptomic profiling of Atlantic salmon skin and head kidney in response to *Lepeophtheirus salmonis* infestation under physiological and elevated temperatures

**DOI:** 10.1016/j.cirep.2026.200285

**Published:** 2026-04-22

**Authors:** Reza Ghanei-Motlagh, Wenlong Cai, Jordan D. Poley, Yuxuan Zhang, Shona K. Whyte, Amber F. Garber, Mark D. Fast

**Affiliations:** a Hoplite Research Lab, Department of Pathology and Microbiology, Atlantic Veterinary College, University of Prince Edward Island, Charlottetown, PEI, Canada; b Huntsman Marine Science Centre, St. Andrews, NB, Canada; c Department of Infectious Diseases and Public Health, Jockey Club College of Veterinary Medicine and Life Sciences, City University of Hong Kong, Kowloon Tong, China; d Onda, Souris, Prince Edward Island, Canada

**Keywords:** Sea lice, Family variation, Family-based transcriptomics, Thermal stress, RNA sequencing, Atlantic salmon, Host resistance

## Abstract

This study explored transcriptomic alterations in the skin and head kidney of Atlantic salmon (*Salmo salar*) from multiple families in response to infestation with the salmon louse (*Lepeophtheirus salmonis*) at the chalimus and adult stages, under elevated (20 °C) versus standard (10 °C) temperature conditions. Family-specific transcriptomic comparisons identified several candidate genes potentially associated with variation in host responses among families to sea lice, as reflected by differential expression patterns between families with varying lice densities. These biomarkers were primarily involved in immune function (e.g., *cd209, cd22, btn2a1, h2-q9, c1qb, muc2, muc5ac, sntx-b, kbas*), tissue repair (e.g., *krt13, tgm2, mmp9, mmp13*), inflammation (e.g., *nlrp1, il1b, nlrc3, nlrp12*), iron homeostasis (e.g., *hbb1, ftm*) and stress response (e.g., *hspa8, herc3*). Under elevated temperature conditions (20 °C), 124 and 324 shared differentially expressed genes (DEGs) were respectively identified in the skin and head kidney of lice-infested salmon. RNA-seq analysis revealed consistent modulation of several thermal stress biomarkers (e.g., *serpinh1, hsp90aa1, hspa8, cirbp, fkbp10*) in both chalimus- and adult-infested salmon under elevated temperature conditions, along with pronounced differences in the diversity of transcriptomic alterations and biological pathways compared to fish parasitized under standard temperature. Our results provide insights into molecular signatures that can help to differentiate more and less susceptible salmon families, supporting the development of selective breeding strategies for improved sea lice management. The temperature-related transcriptomic changes highlighted the climate adaptation strategies adopted by salmon, as well as their potential vulnerability to long-term shifts in water temperature.

## Highlights

• Different families of Atlantic salmon exhibit distinct transcriptional responses to sea lice infestation.
• Family-based transcriptomic analysis holds significant potential for identifying potential molecular biomarkers linked to sea lice resistance/susceptibility in Atlantic salmon.
• Remarkable transcriptomic alterations were observed in Atlantic salmon exposed to elevated (20 °C) compared to standard (10 °C) temperature conditions.
• Both sea lice infestation and thermal stress influenced transcriptional changes in the skin and head kidney of Atlantic salmon.

## Introduction

The salmon aquaculture industry is presently confronted with several challenges, including management of parasitic infestations caused by the salmon louse (*Lepeophtheirus salmonis*), which significantly impact the health and productivity of farmed salmon [1]. The life cycle of sea lice involves eight developmental stages, with the first three larval stages (nauplius I, II and copepodid) that are free-living and planktonic, followed by two sessile stages (chalimus I, II) that attach to the host using a frontal filament, and finally three mobile stages (pre-adult I, II, and adult) that can move freely across the fish and cause progressive tissue damage [2]. There is currently no commercial vaccine protective against sea lice infestation, and the traditional control strategies relying on chemotherapeutants have become less practical due to the increased costs associated with treatment and the emergence of resistant sea lice populations, particularly in regions with intensive production [3]. These limitations have encouraged salmon farming professionals to explore alternative control strategies.

Family-based selective breeding programs are among the most advanced efforts, which prioritize traits such as enhanced growth performance, increased disease resilience, delayed sexual maturation, and improved product quality [4]. Previous studies have shown that sea lice resistance is a polygenic trait that is moderately heritable, suggesting that different salmon families can exhibit varying levels of tolerance to infestation [5]. However, selection for enhanced parasite resistance may involve trade-offs with other desirable traits such as growth performance, tolerance, or infectivity, as resistance can sometimes be genetically correlated with slower growth or altered disease transmission dynamics; therefore, traits related to host-parasite interactions should be optimized in conjunction with overall productivity and sustainability goals [6,7]. Recent studies have identified several candidate genes and pathways involved in the salmon defense mechanisms against sea lice through transcriptomic and genomic approaches, including those linked to immune and inflammatory responses [[8], [9], [10], [11], [12], [13], [14], [15], [16]]. High-throughput transcriptomics, using technologies such as RNA-seq, has emerged as a powerful tool for identifying molecular signatures underlying these responses in fish [12,14]. In addition to applications in selective breeding programs, transcriptomic-associated biomarkers provide promising targets for the design of vaccines and the application of gene editing technologies such as CRISPR-Cas9 [6,17].

The warming of aquatic ecosystems due to global climate change presents significant challenges for both farmed and wild fish populations [18,19]. As poikilothermic organisms, ray-finned fish are particularly sensitive to temperature fluctuations, which influence various aspects of their physiology, including metabolic rate, immune function, growth, haemato-biochemical responses and reproductive performance [20,21]. Elevated temperatures have been reported to intensify the impact of parasitic infections in Atlantic salmon by impairing immune function, altering host-pathogen interactions, and increasing pathogen virulence and host susceptibility to other stressors [[21], [22], [23]]. As the result of the rise in global temperature driven by climate change, understanding the salmon molecular responses to combined challenges of incremental temperature and sea lice infestation remains an important research focus.

More and less susceptible salmon families or individuals are typically classified based on the total number of lice counted per fish [8,13]. Regardless of whether comparisons are made at the family or individual level, variability in responses to infestation is inevitable, as skin tissues collected from specific sites may or may not be equally infested [1,10]. Therefore, both approaches have inherent limitations. However, analyzing data at the family level preserves the genetic diversity represented by individuals from different families, which is crucial for detecting expression patterns that may underlie variation in host responses to sea lice. In our previous study, we conducted a family-based analysis to examine the transcriptomic responses of Atlantic salmon infested with adult compared to chalimus stages of sea lice under varying thermal conditions, highlighting the differences associated with parasite developmental stage [24]. In the present study, we aimed to investigate the transcriptomic responses of the skin and head kidney in Atlantic salmon from the same families following infestation with chalimus and adult stages of sea lice, emphasizing differences associated with family and thermal conditions. By employing an RNA-seq approach, this study aims to advance our understanding of candidate transcriptomic biomarkers that reflect variation in responses among salmon families to sea lice infestation and those associated with thermal stress, with potential applications in selective breeding, gene editing and climate adaptation strategies.

## Materials and methods

### Fish maintenance

Atlantic salmon smolts used in this study originated from 20 families (100 fish per family), produced from a larger breeding program and reared at the Huntsman Marine Science Centre (Huntsman) in St. Andrews, New Brunswick, Canada. The families were generated from 16 sires and 16 dams across three year classes within the breeding program and were expected to exhibit variable performance in sea lice resistance. Some families were minimally genetically related, whereas others were unrelated (Supplementary Data File 1). All fish (16.5 g mean weight) were anesthetized with tricaine methane sulphonate (MS-222, 150 mg/l), individually identified with a passive integrated transponder (PIT) tag and reared communally in a partial recirculating freshwater system, under ambient light conditions and water temperature maintained 1–3 °C above ambient. At smolt size (> 50 g), fish were vaccinated and subsequently smolted into full strength seawater in a single 7.5 m^3^ tank [24]. During this period, fish were fed a commercial diet (Skretting, Canada). Weight was recorded at the post-smolt stage to allow allocation into eight fiberglass tanks (1.3 m^3^) containing seawater, with comparable biomass maintained in each tank (6 fish per family, 120 fish per tank). Due to COVID-19 constraints in sourcing sea lice for the challenge, fish were held at 2–6 °C for approximately 6 months with maintenance feeding, after which the salmon were gradually acclimated to ambient temperature. Fish were held at 10 ± 1 °C for a 10-day acclimation period, and then the temperature in four of the eight tanks was gradually increased by 1 °C per day until reaching 20 ± 1 °C [24].

### Experimental challenge, lice counting and tissue collection

The sea lice challenge, parasite enumeration and tissue sampling were conducted as previously described [24]. Briefly, post-smolt Atlantic salmon were exposed to 100 copepodids per fish for 1 h at either 10 °C or 20 °C. Sampling was conducted at two developmental stages of *L. salmonis* (110 degree days when lice reached the chalimus II stage, and 360 degree days when adult lice were present). At each time point, all fish from two tanks per temperature were euthanized individually with an overdose of MS-222 (250 mg/l) followed by examination. Body weight (BW) and fork length (FL) were recorded for each fish, and condition factor (CF) was calculated as data was recorded: CF = [100 × BW (g)] / FL^3^ (cm). Lice counts were performed on all body surfaces of each fish, including the gills, which were removed and examined under a dissecting microscope during the chalimus stage. Immediately after these assessments, two tissue samples were consistently collected from each individual fish. These included a skin sample (∼ 10 mm × 10 mm) taken from the area posterior to the dorsal fin, a common site for parasite attachment, and a head kidney sample (right portion). Fish were handled in this manner to ensure tissue preservation within 20 min post-euthanasia. All samples were individually preserved in RNAlater stabilization solution for subsequent molecular analyses. Lice density was calculated individually for both chalimus- and adult-infested fish using lice counts and body weight, following the equation provided by Gjerde et al. [25], and family-level averages were subsequently determined. All animal procedures were conducted in accordance with protocols approved by the UPEI Animal Care Committee (Protocol #16–051) and the Fisheries and Oceans Canada–Huntsman Marine Science Centre Regional Animal Care Committee (AUP #20–31).

### RNA preparation and sequencing

Total RNA was isolated using TRIzol-chloroform phase separation and subsequently treated with DNase I using a commercial kit (Zymo Research, CA, USA) [24]. RNA samples were quantified using a NanoDrop spectrophotometer (ND 2000, Thermo Scientific), and RNA integrity was assessed using 1.5 % agarose gel electrophoresis [26]. A total of 482 extractions (241 for each tissue) were completed, and pure RNA (A260/A280 ≥ 1.9 and A260/A230 ≥ 1.7) was achieved for all samples. RNA-seq libraries were prepared using the TruSeq Stranded mRNA Library Prep Kit (Illumina, CA, USA) and sequenced as previously described [24].

All 20 families were assessed for parasite enumeration at 10 °C or 20 °C. Tissue samples were collected from 12 families, including four maintained at 10 °C (F153, F154, F185, F265), four at 20 °C (F215, F292, F397, F415) and four at both temperatures (F175, F229, F361, F419). Of the latter, families F175, F361 and F419 were included in the RNA-seq analysis to enable within-family comparisons across temperature conditions. Selection of the remaining families for RNA-seq analysis was based on distinct lice density profiles observed in fish infested with adult *L. salmonis* at 10 °C, as lice densities varied among families depending on parasite developmental stage and temperature. This approach also aimed to better reflect conditions observed under natural infestations. Accordingly, F229 was excluded due to overlapping lice densities with F175 and F419, and representatives from F265 at 10 °C and F292 at 20 °C were included to maintain balanced representation across temperature conditions (see Supplementary Table 1 for details). A total of 3–5 samples per family were included for RNA-seq analyses. The families selected for RNA-seq analysis were genetically unrelated (Supplementary Data File 1).

### RNA-seq and gene ontology (GO) enrichment analyses

FastQC v0.15.3 was used for quality control of the raw sequence (fastq) data. Residual adaptor sequences and low-quality bases were filtered out using Trimmomatic v0.36 as described previously [27]. Trimmed reads were aligned to the Atlantic salmon genome assembly (Ssal_v3.1, GenBank accession no. GCF_905237065.1) using STAR v2.7.9a, which also generated and sorted the resulting BAM files [14]. Count matrices were generated using featureCounts, included in the Subread package v1.6.5 [28], to quantify reads mapped to individual genes. Differential expression analysis between groups (families) differing in lice density (3–5 samples per family) and temperature conditions was conducted using the edgeR package. As samples collected from individuals in 2 of the 5 families included in the RNA-seq analysis (i.e. F265 and F292) were assessed only at either 10 °C or 20 °C, temperature-based pairwise comparisons (20 °C vs. 10 °C) were performed both within individual families (for 3 families with representatives at both temperatures, i.e., F419, F361 and F175; 3–5 samples per family) and across all families combined (15–20 samples per temperature), following infestation with adult or chalimus stages. The selection criteria for DEGs identified in this study were as follows: |log_2_-fold change| > 1.25, adjusted *p*-value < 0.05 and |log of counts per million reads| > 1.00 [24]. Principal component analysis (PCA) was employed to identify variability among RNA-seq samples for each pairwise comparison, using raw count data from the complete set of expressed genes. Hierarchical clustering of shared DEGs across various pairwise comparisons was carried out using the “pheatmap” function in R. Across all RNA-seq samples, the mean read-assignment rate was ∼65 % in the head kidney and ∼62 % in the skin. The number of detected genes (≥ 1 read) averaged 36,000–38,000 per library, and approximately 25,000–27,000 genes showed expression levels above 1 CPM (counts per million). Each sample (head kidney or skin) contained ∼30 ± 5 million reads, indicating sufficient depth and coverage for transcriptome analyses performed.

Gene annotation was conducted using AnnotationHub with the *Salmo salar* database (no. AH114250). Fully annotated DEGs were subjected to GO and Kyoto Encyclopedia of Genes and Genomes (KEGG) enrichment analyses using ClueGO plug-in (v2.5.10) in Cytoscape v3.10.2 [29,30]. ClueGO enrichment analysis was performed with the following options and adjustments: pathway *p*-value cut-off < 0.05, kappa score: 0.4 and right-sided hypergeometric test with Benjamini-Hochberg *p* value correction [24]. Enriched GO terms were grouped based on kappa-score similarity of associated genes. Within each functional group, the most statistically significant term is identified as the leading term and is highlighted in the network. Other significantly enriched terms in the same group are considered sub-leading. The enriched (over-represented) leading GO terms identified from temperature-related comparisons (including both pairwise and shared DEGs) were categorized into distinct functional themes based on their parent GO terms as well as DEGs and sub-leading GO terms involved in each GO group. The GO and KEGG databases used for grouping the leading GO terms were in accordance with those previously described [24,31].

### Selection of putative genes linked to family differences in response to sea lice infestation

To minimize the inclusion of expression differences unrelated to lice density, a combination of the following selection criteria was applied: genes were prioritized based on (*i*) repeated detection across multiple pairwise comparisons between families infested with chalimus or adult stages of sea lice at 10 °C and/or 20 °C, (*ii*) functional relevance mainly to immune response, inflammation or tissue repair, and (*iii*) consistent expression differences between families exhibiting lower and higher lice densities in either skin or head kidney. Candidate genes typically showed a fold change > ±3, occurred in at least three of the twenty-four pairwise comparisons per tissue (often in ≥ five), and were expressed in either or both tissues. Functional annotation further emphasized immune-related transcripts likely modulated by infestation. Only genes displaying reproducible expression trends between low- and high-lice families were retained and discussed as potential biomarkers associated with host responses to sea lice.

### Quantitative real-time PCR (qPCR) analyses

qPCR assays included all head kidney and skin tissue samples from 12 families for which tissue collection was performed. The family distribution across temperature conditions (10 °C and 20 °C) were as follows: F153, F154, F185, F265 at 10 °C; F215, F292, F397, F415 at 20 °C; F175, F229, F361, F419 at both temperatures [24]. qPCR analyses were performed to assess the expression of the following genes: cluster of differentiation 83 (*cd83*), c-type lectin domain family 4 (*clec4*), collagen alpha-1(I) chain (*col1a1*), matrix metalloproteinase 9 (*mmp9*), neutrophil cytosolic factor 2 (*ncf2*), serum amyloid A5 (*saa5*), tissue inhibitor of metalloproteinase 2 (*timp2*) and thioredoxin b (*txnb*) serving as genes of interest (GOIs), and eukaryotic translation initiation factor 3 subunit 6 (*eif3*), elongation factor 1 alpha (*ef1a*) and RNA polymerase I (*rpl1*) selected as housekeeping genes [[32], [33], [34], [35], [36], [37], [38], [39], [40]]. These genes were selected based on previous studies that showed their modulation in response to sea lice infestation (all GOIs tested) and/or thermal stress (*mmp9* and *txnb*) [24,35,36,38]. The selected genes, along with the primer sequences used for qPCR, are provided in Supplementary Data File 1. Primer concentrations were optimized individually for each target to ensure optimal amplification efficiency and specificity.

Complementary DNA (cDNA) synthesis was performed using 1 µg of total RNA per sample in 20 µl reaction volume using the High Capacity cDNA Reverse Transcription Kit (Applied Biosystems, CA, USA), following the manufacturer’s instructions. The stock cDNA samples were individually diluted to achieve 1:20 or 1:200 ratios prior to running qPCR, where 1:20 diluted cDNA was used for *saa5, clec4, cd83* and *ncf2* (only for skin) and 1:200 dilutions were applied in other gene expression assays. The SYBR-based qPCR assays were specific to the tested GOIs and reference genes. qPCR reactions were performed on CFX96 Touch Real-Time PCR System (BioRad) in 12 µl reaction mixture composed of 5 µl of Sso Advanced™ Universal SYBR® Green Supermix (Bio-Rad), 0.5 µl of 10 mM forward and reverse primers, 4 µl of nuclease-free water and 2 µl of cDNA template using the cycling conditions: 95 °C for 30 s, 95 °C for 15 s, 60 °C for 15 s, for 39 cycles, followed by a melt curve analysis ramping from 65 to 95 °C, with fluorescence detected every 0.5 s at a ramp rate of 0.5 °C. Standard curves for all genes were separately generated for the head kidney and skin samples, using a minimum of 5-point serial dilutions with linearity (R^2^) greater than 0.970 and amplification efficiency ranging between 90 % and 105 % (Supplementary Data File 1). qPCR reactions were run in triplicate, along with a calibrator pool (tissue-specific equimolar pool of all samples) and two control reactions (no-template control and no reverse transcriptase control). No reverse transcriptase controls were run using pools of 48 samples per reaction. Negative control reactions occasionally showed amplification; however, the observed amplification was > 7 cycles behind relevant samples and outside the range of linearity for the assay. Likewise, none of the negative control amplifications were repeatable within acceptable standard deviations. Cycle threshold (C_T_) values were analyzed using geNorm to calculate the M-values. All GOIs were normalized to the geometric means of housekeeping genes (*eif3, ef1a, rpl1*) with incorporation of the amplification efficiencies and calibrated against the calibrator pool to produce the relative quantification (RQ) values. The RQ values were log_2_-transformed and statistically compared across temperature and families.

For qPCR quality control, a total of 2651 and 2541 reactions were respectively completed in triplicate for the head kidney and skin samples. Standard deviations (SD) below 0.50 for technical replication were obtained for 98.4 % and 96.5 % of the reactions related to head kidney and skin, respectively. Reference genes were stably expressed across head kidney (GeNorm M-value of 0.432 [*eif3*|*rpl1*] and 0.504 [*eif3*|*ef1a*] with ΔCt standard deviations of 0.44, 0.53, and 0.54 for *eif3, rpl1* and *ef1a*, respectively) and skin (GeNorm M-value of 0.311 [*eif3*|*ef1a*] and 0.426 [*eif3*|*rpl1*] with ΔCt standard deviations of 0.38, 0.42, and 0.48 for *eif3, rpl1* and *ef1a*, respectively) samples. The correlation in expression of the three reference genes was significant (*p* < 0.0001) with Pearson’s *r*-values of 0.90 (*eif3*|*ef1a*), 0.87 (*eif3*|*rpl1*) and 0.80 (*rpl1*|*ef1a*) for head kidney and 0.98 (*eif3*|*ef1a*), 0.96 (*eif3*|*rpl1*), and 0.94 (*rpl1*|*ef1a*) for skin.

### Statistical analysis

The normality of growth and gene expression data was checked using the Shapiro-Wilk test. One-way analysis of variance (ANOVA) followed by Tukey’s *post hoc* test (for family-based differences) and independent student’s *t*-test (or Mann-Whitney U test, for temperature-based differences) were used to determine significant differences between the mean values. The growth and q-PCR data analyses were conducted using the GraphPad Prism v10 (GraphPad Software Inc, USA).

Relationships between lice abundance [24] and BW were evaluated using both family-level and within-family approaches. Lice abundance, obtained from our previous study [24], was used as the primary measure of parasite burden, as lice density is a derived metric that incorporates body weight in its calculation and may introduce circularity when assessing relationships with growth traits. At the family level, PCA and Pearson correlation analyses were performed using family mean values. PCA was conducted in R using the prcomp function on centered and scaled variables, and results were visualized using biplots to illustrate sample distribution and variable contributions. In addition, within-family correlation analyses were performed to assess associations between lice abundance and BW at the individual level within each family. Correlation analyses were conducted separately for each temperature (10 °C and 20 °C) and parasite developmental stage (adult and chalimus) to account for condition-specific variation. Pearson correlation coefficients (*r)* and associated *p*-values were calculated using the cor.test function in R.

## Results

### Lice density variations

**Fig. 1:**
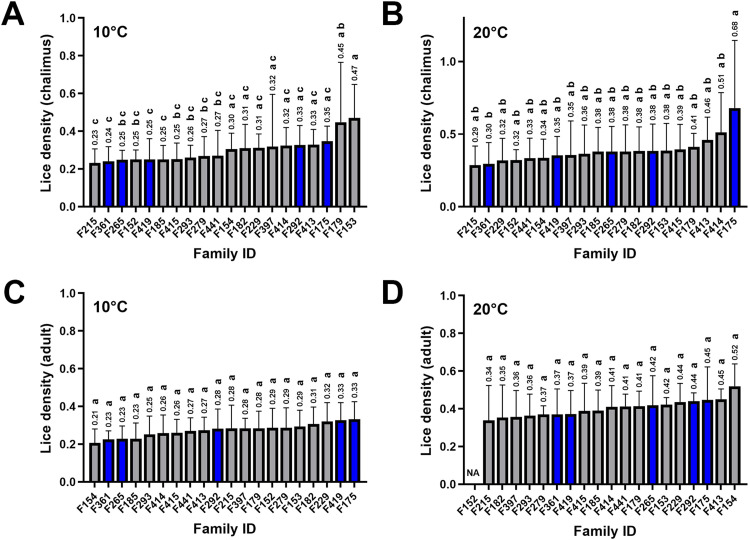
Summary of mean lice density (MLD; mean ± SD) in salmon families infested with chalimus or adult stages under physiological (10 °C) and elevated (20 °C) temperature conditions. (**A**) MLD in chalimus-infested families at 10 °C. (**B**) MLD in chalimus-infested families at 20 °C. (**C**) MLD in adult-infested families at 10 °C. (**D**) MLD in adult-infested families at 20 °C. Different lowercase letters denote significant differences (*p* < 0.05) in MLD among families. MLD values are displayed above the bars. Blue-highlighted bars indicate families selected for RNA-seq analysis.

Across all 20 families, the mean lice density (MLD) exhibited greater variability in chalimus-infested families compared to adult-infested families under both temperature conditions (Fig. 1). Based on the results, MLD values (chalimus or adult) for families infested at 10 °C or 20 °C (selected for RNA-seq) were respectively as follows (see Supplementary Table 2 for details): MLD-T10C, F175 (0.347) > F292 (0.327) > F419 (0.250) > F265 (0.248) > F361 (0.239); MLD-T20C, F175 (0.680) > F292 (0.384) > F265 (0.380) > F419 (0.352) > F361 (0.295); MLD-T10A, F175 (0.331) > F419 (0.326) > F292 (0.281) > F265 (0.228) > F361 (0.225); MLD-T20A, F175 (0.447) > F292 (0.440) > F265 (0.419) > F419 (0.371) > F361 (0.370). One-way ANOVA revealed a significant difference in MLD among families infested with the chalimus stage at 10 °C (*p* = 0.0002), with the Tukey post hoc test showing significant pairwise differences in 14 out of 190 comparisons (*p* < 0.05). At 20 °C, one-way ANOVA did not indicate an overall significant difference among families infested with the chalimus stage; however, the Tukey test identified one significant pairwise comparison (*p* < 0.05). For families infested with the adult stage at 10 °C, ANOVA indicated a significant overall difference in MLD (*p* = 0.0474), although no significant pairwise differences were detected using the Tukey test (*p* > 0.05). At 20 °C, neither ANOVA (*p* = 0.9309) nor the Tukey test (*p* > 0.05) revealed significant differences among families infested with the adult stage.

MLD values were predominantly higher in both chalimus- and adult-infested salmon families at 20 °C compared to 10 °C, with significant differences (*p* < 0.05) observed in 6 out 20 comparisons for chalimus-infested families and 14 out of 19 comparisons for adult-infested families (Supplementary Fig. 1). The higher lice density in infested salmon exposed to elevated temperature may be associated with increased activity and host-seeking behaviour of copepodids at elevated temperatures, leading to enhanced parasite settlement. Additionally, despite equal experimental challenge doses, elevated temperature can accelerate lice development while also altering salmon stress and immune responses [19].

### Growth parameters in lice-infested families

BW, FL and CF varied among families infested with chalimus or adult stages at different temperatures (Supplementary Figs. 2–4). Among the families included in the RNA-seq analysis, mean differences were not statistically significant in most cases (*p* > 0.05). Comparisons between temperature conditions revealed more consistent patterns, with BW, FL and CF generally lower in both chalimus- and adult-infested families at higher temperatures in most cases (Supplementary Fig. 1). These differences were, however, more pronounced and statistically significant (*p* < 0.05) in families infested with adult stages.

### Relationships between parasite burden and host size

PCA based on family-level mean values of lice abundance and BW showed temperature-driven separation of samples, with BW and lice abundance contributing along largely independent axes. Family-level Pearson correlation analyses indicated moderate positive relationships between lice abundance and BW, particularly during adult infestations (*r* = 0.63–0.66, *p* < 0.01), consistent with host-size effects. In contrast, family-wise analyses showed that correlations were predominantly positive but generally weak and often not statistically significant, indicating no consistent within-family association between parasite burden and growth and limited evidence for trade-offs (Supplementary Fig. 5).

### Transcriptomic responses and GO/KEGG enrichment analyses of DEGs

#### DEGs and GO terms/KEGG pathways associated with family differences

Pairwise comparisons between families of different lice density were performed for a specific infestation stage at a particular temperature for each tissue (T10A, T10C, T20A and T20C; 6 pairwise comparisons between 4 families infested with adult or chalimus stages of lice at 10 °C or 20 °C; 24 pairwise comparisons per tissue, see Supplementary Data Files 2–10). The number of DEGs identified based on each pairwise comparison was highly variable in both skin and head kidney tissues (T10A, 61–490 in the head kidney & 226–2687 in the skin; T10C, 196–433 in the head kidney & 223–395 in the skin; T20A, 141–386 in the head kidney & 98–1539 in the skin; T20C, 2–399 in the head kidney & 183–450 in the skin), and there were no shared DEGs among multiple comparisons related to T10A, T10C, T20A or T20C (Table 1, Supplementary Data Files 2–9). As shown in Supplementary Fig. 6 and Supplementary Fig. 7, PCA plots generated using the gene expression data associated with family-based comparisons within T10A, T10C, T20A or T20C resulted in a distinct or moderately distinct clustering (*p* < 0.05 for PC1 or PC2) of skin samples related to fish infested by the adult stages at 10 °C (T10A) and head kidney samples of fish parasitized with chalimus stages of lice (T10C) in most cases. However, skin and head kidney samples collected from fish infested at 20 °C (chalimus or adult) were not clearly segregated in most comparisons (*p* > 0.05 for PC1 or PC2) (Supplementary Fig. 6 and Supplementary Fig. 7). In addition, individual samples associated with specific families were found to be more scattered in several cases, suggesting variations among representatives belonging to each family in response to infestation with chalimus/adult stages of lice at different temperatures.

**Table 1:** Differentially expressed genes (DEGs) identified based on pairwise comparisons between salmon families with different lice burden (parasitized with chalimus or adult stages of lice) under a particular temperature for each tissue.

Pairwise comparisons	No. of DEGs in skin			No. of DEGs in head kidney		
	Up	Down	Total	Up	Down	Total
T10A						
F265 vs. F175	117	109	226	39	65	104
F361 vs. F175	1881	686	2567	121	63	184
F265 vs. F361	659	1859	2518	114	376	490
F175 vs. F419	186	313	499	33	28	61
F265 vs. F419	201	216	417	58	121	179
F361 vs. F419	1956	731	2687	122	100	222
Shared DEGs			0			0
T10C						
F265 vs. F175	151	244	395	85	196	281
F361 vs. F175	108	183	291	238	159	397
F265 vs. F361	113	143	256	144	289	433
F175 vs. F419	149	74	223	78	118	196
F265 vs. F419	123	152	275	113	183	296
F361 vs. F419	161	214	375	119	114	233
Shared DEGs			0			0
T20A						
F292 vs. F175	178	130	308	63	78	141
F361 vs. F175	368	214	582	69	120	189
F361 vs. F292	930	609	1539	98	93	191
F175 vs. F419	37	61	98	158	174	332
F292 vs. F419	122	112	234	123	114	237
F361 vs. F419	146	98	244	162	224	386
Shared DEGs			0			0
T20C						
F292 vs. F175	89	94	183	5	15	20
F361 vs. F175	207	101	308	7	35	42
F361 vs. F292	258	94	352	0	2	2
F175 vs. F419	134	63	197	315	84	399
F292 vs. F419	165	68	233	159	36	195
F361 vs. F419	344	106	450	78	65	143
Shared DEGs			0			0

The results of GO analysis of DEGs identified based on pairwise comparisons between different families are provided in Supplementary Data Files 2–9. In the head kidney (Supplementary Data Files 4–7), several enriched GO terms were associated with general stress responses and immune-related processes (“killing of cells of another organism”, T10A and T10C; “ATP-dependent protein folding chaperone”, T10C; “response to transforming growth factor beta” and “humoral immune response”, T20A). Enrichment of multiple GO terms/KEGG pathways (e.g., “ferroptosis”, “cellular detoxification” and “hemoglobin complex”) containing ferritin (middle subunit) and/or hemoglobin subunits (*hba, hba1, hba2, hba4, hbb* or *hbb1*) was a noticeable finding for the majority of pairwise comparisons in the head kidney and skin. GO terms/KEGG pathways enriched in the skin of families of different lice densities (Supplementary Data Files 6–9, Table 2) were associated with ‘immune-inflammatory response’, ‘cell signaling’, ‘tissue repair/wound healing’, ‘response to stimulus, damage response and cellular process’, ‘metabolic process/pathway’, ‘antioxidant activity and redox reaction’ and ‘transport and binding’.

**Table 2:** Selected GO terms/KEGG pathways enriched in the skin of infested families with varying lice densities.

Category	GO/KEGG ID	GO terms/KEGG pathways	Corresponding pairwise comparisons
Immune-inflammatory response	KEGG:04,145; GO:0006,935; GO:0032,587	“phagosome”, “chemotaxis” and “ruffle membrane”	T10A (F361 vs. F175), T10A (F265 vs. F361), T10A (F361 vs. F419) and/or T20A (F361 vs. F292)
	GO:0008,009	“chemokine activity”	T10A (F361 vs. F175), T10A (F265 vs. F361), T10A (F175 vs. F419), T10A (F265 vs. F419), T10A (F361 vs. F419), T20A (F292 vs. F175), T20A (F361 vs. F175) and T20A (F361 vs. F292)
	GO:0001,816	“cytokine production”	T10A (F361 vs. F419), T20A (F361 vs. F175) and T20A (F361 vs. F292)
	GO:0001,846; GO:0001,849	“opsonin binding” and “complement component C1q complex”	T10A (F361vs. F175)
	GO:0006,954	“inflammatory response”	T10A (F175 vs. F419), T10A (F361 vs. F419) and T20A (F361 vs. F292)
	KEGG:04,672	“intestinal immune network for IgA production”	T10A (F265 vs. F175) and T10A (F361 vs. F419)
	GO:0042,110	“T cell activation” and “B cell activation”	T10A (F361 vs. F419) and/or T20A (F361 vs. F175)
	GO:0042,742; GO:0005,044; GO:0006,959	“defense response to bacterium”, “scavenger receptor activity” and “humoral immune response”	T10A (F265 vs. F361), T10A (F361 vs. F419), T10C (F361 vs. F419) and/or T20A (F361 vs. F175)
	GO:0051,607	“defense response to virus”	T20A (F292 vs. F175) and T20A (F292 vs. F419)
	GO:0042,554	“superoxide anion generation”	T20A (F292 vs. F175), T20A (F361 vs. F292) and T20C (F361 vs. F292)
Cell signaling	KEGG:04,115; KEGG:04,068; GO:0097,720; GO:0007,178; GO:0007,165; GO:0005,102; KEGG:04,350; GO:0030,215; GO:0007,229	“p53 signaling pathway”, “FoxO signaling pathway”, “calcineurin-mediated signaling”, “cell surface receptor protein serine/threonine kinase signaling pathway”, “signal transduction”, “signaling receptor binding”, “TGF-beta signaling pathway”, “semaphorin receptor binding” and “integrin-mediated signaling pathway”	T10A (F361 vs. F175), T10A (F265 vs. F361) and T10A (F361 vs. F419)
	GO:0038,127	“ErbB signaling pathway”	T10A (F175 vs. F419), T10A (F265 vs. F419) and T20A (F361 vs. F292)
	KEGG:04,620	“Toll-like receptor signaling pathway”	T10A (F265 vs. F419) and T20A (F361 vs. F292)
	GO:0043,409	“negative regulation of MAPK cascade”	T10A (F175 vs. F419) and T20A (F361 vs. F175)
	KEGG:04,010; KEGG:04,150; KEGG:04,625; GO:0007,219; GO:0070,371; GO:0007,259	“MAPK signaling pathway”, “mTOR signaling pathway”, “C-type lectin receptor signaling pathway”, “Notch signaling pathway”, “ERK1 and ERK2 cascade” and “cell surface receptor signaling pathway via JAK-STAT”	T10A (F361 vs. F419)
	GO:0035,567	“non-canonical Wnt signaling pathway”	T20A (F292 vs. F175)
	GO:0019,207	“kinase regulator activity”	T10A (F361 vs. F175), T10A (F265 vs. F361), T10A (F361 vs. F419) and T20A (F361 vs. F292)
	GO:0005,942	“phosphatidylinositol 3-kinase complex”	T10A (F361 vs. F175), T10A (F265 vs. F361), T10A (F361 vs. F419), T10C (F265 vs. F175) and T10C (F361 vs. F175)
	GO:0048,013; KEGG:04,010; KEGG:04,115; KEGG:04,912; GO:0007,266; KEGG:04,370; KEGG:04,625; KEGG:04,621; KEGG:04,622; GO:0007,229; GO:0007,166	“ephrin receptor signaling pathway”, “MAPK signaling pathway”, “p53 signaling pathway”, “GnRH signaling pathway”, “Rho protein signal transduction”, “VEGF signaling pathway”, “C-type lectin receptor signaling pathway”, “NOD-like receptor signaling pathway”, “RIG-I-like receptor signaling pathway”, “integrin-mediated signaling pathway” and “cell surface receptor signaling pathway”	T20A (F361 vs. F292)
Tissue repair/wound healing	GO:0003,810; GO:0008,544; GO:0030,216	“protein-glutamine gamma-glutamyltransferase activity”, “epidermis development” and “keratinocyte differentiation”	T10A (F175 vs. F419), T10A (F265 vs. F419), T10C (F361 vs. F419), T20A (F361 vs. F175), T20A (F361 vs. F419) and/or T20A (F361 vs. F292)
	GO:0030,574; GO:0008,237; GO:0030,198; GO:0030,312; GO:0062,023; GO:0004,857; GO:0099,513; GO:0045,111; GO:0001,944	“collagen catabolic process”, “metallopeptidase activity”, “extracellular matrix organization”, “external encapsulating structure”, “collagen-containing extracellular matrix”, “enzyme inhibitor activity”, “polymeric cytoskeletal fiber”, “intermediate filament cytoskeleton” and “vasculature development”	T10A (F361 vs. F175), T10A (F265 vs. F361), T10A (F361 vs. F419) and/or T20A (F361 vs. F292)
	GO:0030,020	“extracellular matrix structural constituent conferring tensile strength”	T10A (F361 vs. F175), T10A (F265 vs. F361), T10A (F361 vs. F419), T10C (F361 vs. F419), T20A (F361 vs. F292) and T20C (F292 vs. F419)
	GO:0005,518	“collagen binding”	T10A (F361 vs. F175), T10A (F265 vs. F361) and T10A (F361 vs. F419)
	GO:0005,178; GO:0008,201	“integrin binding” and “heparin binding”	T10A (F175 vs. F419), T10A (F265 vs. F361), T10A (F361 vs. F175), T10A (F361 vs. F419), T20A (F361 vs. F175) and/or T20A (F361 vs. F292)
	GO:0015,631	“tubulin binding”	T10A (F361 vs. F175) and T10A (F361 vs. F419)
	KEGG:04,820	“cytoskeleton in muscle cells”	T10A (F361 vs. F175), T10A (F265 vs. F361), T10A (F361 vs. F419), T10C (F265 vs. F175), T10C (F361 vs. F175), T10C (F175 vs. F419), T20A (F361 vs. F292), T20C (F361 vs. F292) and T20C (F361 vs. F419)
	GO:0016,460; GO:0043,292	“myosin II complex” and “contractile muscle fiber”	T10C (F265 vs. F175), T10C (F361 vs. F175), T10C (F175 vs. F419), T20C (F361 vs. F175), T20C (F361 vs. F292) and T20C (F361 vs. F419)
	GO:0061,041	“regulation of wound healing”	T10C (F265 vs. F419), T20A (F361 vs. F175) and T20A (F361 vs. F292)
	GO:0005,861	“troponin complex”	T10C (F265 vs. F175), T10C (F361 vs. F175), T20C (F361 vs. F175), T20C (F361 vs. F292) and T20C (F361 vs. F419)
Response to stimulus, damage response and cellular process	GO:0140,662; GO:0009,266; GO:0005,832	“ATP-dependent protein folding chaperone”, “response to temperature stimulus” and “chaperonin-containing T-complex”	T10A (F361 vs. F175), T10A (F361 vs. F419), T10C (F361 vs. F175), T10C (F265 vs. F361), T10C (F361 vs. F419), T20A (F361 vs. F292) and/or T20C (F361 vs. F419)
	GO:0006,974; GO:0006,281; GO:0000,075	“DNA damage response”, “DNA repair” and “cell cycle checkpoint signaling”	T10A (F361 vs. F175), T10A (F361 vs. F419) and/or T10A (F265 vs. F361)
	KEGG:04,210	“apoptosis”	T10A (F361 vs. F419) and T20A (F361 vs. F292)
	KEGG:04,218	“cellular senescence”	T10A (F361 vs. F175), T10A (F265 vs. F361), T10A (F361 vs. F419) and T20A (F361 vs. F292)
Metabolic process/pathway	KEGG:00,561; GO:0006,575	“glycerolipid metabolism” and “amino acid metabolic process”	T10A (F361 vs. F175), T10A (F265 vs. F361) and/or T10A (F361 vs. F419)
	KEGG:00,590; GO:0006,693	“arachidonic acid metabolism” and “prostaglandin metabolic process”	T10A (F265 vs. F419), T10A (F175 vs. F419), T10A (F361 vs. F419), T20A (F361 vs. F175), T20A (F292 vs. F419) and T20A (F361 vs. F292)
	GO:0006,082; GO:0019,752	“organic acid metabolic process” and “carboxylic acid metabolic process”	T10A (F265 vs. F361), T10A (F361 vs. F419) and T20A (F361 vs. F292)
	GO:0072,593	“reactive oxygen species metabolic process”	T10A (F265 vs. F175), T10A (F265 vs. F361), T20A (F292 vs. F175), T20A (F361 vs. F175) and T20A (F361 vs. F292)
	KEGG:00,010; GO:0006,090; GO:0019,362	“glycolysis/gluconeogenesis”, “pyruvate metabolic process” and “pyridine nucleotide metabolic process”	T10C (F361 vs. F175), T10C (F175 vs. F419), T10C (F265 vs. F175), T20C (F292 vs. F175), T20C (F361 vs. F292), T20C (F175 vs. F419) and/or T20C (F361 vs. F419)
Antioxidant activity and redox reaction	GO:0004,364	“glutathione transferase activity”	T10A (F361 vs. F175) and T10A (F361 vs. F419)
	GO:0005,507	“copper ion binding”	T10A (F361 vs. F175), T10A (F265 vs. F361) and T10A (F361 vs. F419)
Transport and binding	GO:0020,037	“heme binding”	T10A (F265 vs. F175), T10A (F265 vs. F361) and T20A (F361 vs. F292)
	GO:0032,553; GO:1901,363	“ribonucleotide binding” and “heterocyclic compound binding”	T10A (F361 vs. F175) and T10A (F265 vs. F361)
	GO:0043,167	“ion binding”	T10A (F361 vs. F175), T10A (F265 vs. F361) and T10A (F361 vs. F419)

The more frequent and/or significant DEGs obtained based on pairwise comparisons between families of different lice density (24 comparisons per tissue) were found to primarily belong to DNA damage/apoptosis, cell adhesion, signal transduction/inflammation, iron/platelet homeostasis, stress/heat shock responses, tissue repair/cytoskeletal organization and innate/adaptive immune responses (see Table 3). Representative genes showing the most pronounced transcriptional changes in family-based comparisons across tissues and temperatures included *hba4, hbb1, psap, prgr1, adgre2, dmbt1, tnfrsf19* and *iglc1* in the head kidney; *rab6b, btn2a1, cd209d, aloxe3, trpc3, gimap8, krt13, rock2, zg16* and *cxcl2* in the skin; and *nlrc3, nlrp1, nlrp3, nlrp5, nlrp12, icn, c1qb, trim47, anxa5* and *kbas* in both tissues.

**Table 3:** List of more abundant and/or significant biomarkers identified based on pairwise comparisons (24 per tissue) between families with varying lice densities.

Category	Gene symbol	
	Head kidney	Skin
DNA damage/apoptosis	*ppp2r2a, banf1, trim39*	*casp8, ddx17, herc3, pla2r1, plekhn1, usp7, banf1, trim39*
Cell adhesion	—	*ceacam20, ephb6, lama3, lamb3, lgals4, spon2b*
Signal transduction/inflammation	*ptgr1, serpinb1, nlrc3, nlrp1, nlrp12, nlrp3*	*card14, cmklr1, ltb4r, nlrc5, peli1, pla2g2a, pla2g4c, pld4, ptgs2, ptgs2b, rock2, serpinb1, nlrc3, nlrp1, nlrp12, nlrp3*
Iron/platelet homeostasis	*snaclec1, hba1, hba4, hbb, hbb1, tfa, ftm*	*tf2, hebp1, hebp2, snaclec1, hba1, hba4, hbb, hbb1, tfa* and *ftm*
Stress/heat shock responses	*dnajb5, hsp90aa1, hspa8*	*hspa14, hspd1, dnajb5, hsp90aa1, hspa8*
Tissue repair/cytoskeletal organization	*ccn2*	*adamts12, aloxe3, ccn2, hbegf, igfbp5, krt13, krt18, krt18a, krt19, krt20, krt8, krt8.2.l, krt9, col1a1, col1a2, col6a1, col6a2, col6a6, col14a1, col18a1, col21a1, col28a1, col10a1, col11a2, mmp9, mmp13, tgfb3, tgm1, tgm2, tgm3, timp2, timp3, tnik*
Innate/adaptive immune responses	*adgre2, cd152, cd163, cd209, cd244, cd79a, dmbt1, fcgr3, gimap6, igkv1–39, igkv4–1, iglc1, iglc2, k29–213, nkl, pglyrp2, pigr, prf1.3, timd4, anxa5, c1qb, cd209c, cd209d, cd209e, cd22, cd55, clec4m* [*cd209l*], *clec4e* [*mincle*], *ctsc, fos, h2-q9, kbas, litaf, mr1, palm3, psap, psmb9a, rap1b, samd9, sntx-b, tap2b, siglec5, trim16, trim25, trim47*	*adgrf3, aerolysin-like protein* [no symbol], *arg1, btn1a1, btn2a1, btn3a2, c3, camp, ccl20, ccl21, ccl25a, ccl4, cd2, cd200, cd300ld, cd9, clec10a* [*mgl*], *cxcl11, cxcl2, cxcr1, cxcr4, dnase1l3, dusp1, dusp4, dusp5, epx, erbb3, fcgbp, gimap8, ifi44, ighd, igkc, il12rb1, il1b, il21r, integumentary mucin C.1-like* [no symbol], *intestinal mucin-like protein* [no symbol], *irf7, isg15, itln1, jun, lcn, lect2, mal, mopc-321, mrc1, mrc2, muc2, muc5ac, myb, nfatc2, nos2b, pbx1, pigr, prdm1, rab6b, rnd1, saa5, samhd1, sema3b, sema3d, sema3f, sema7a, socs2, socs3, stat1, tnfrsf14, tnfrsf19, traf2, anxa5, c1qb, cd209c, cd209d, cd209e, cd22, cd55, clec4m* [*cd209l*], *clec4e* [*mincle*], *ctsc, fos, h2-q9, kbas, litaf, mr1, palm3, psap, psmb9a, rap1b, samd9, sntx-b, tap2b, siglec5, trim16, trim25, trim47*

#### DEGs and GO terms/KEGG pathways related to temperature increment

**Fig. 2:**
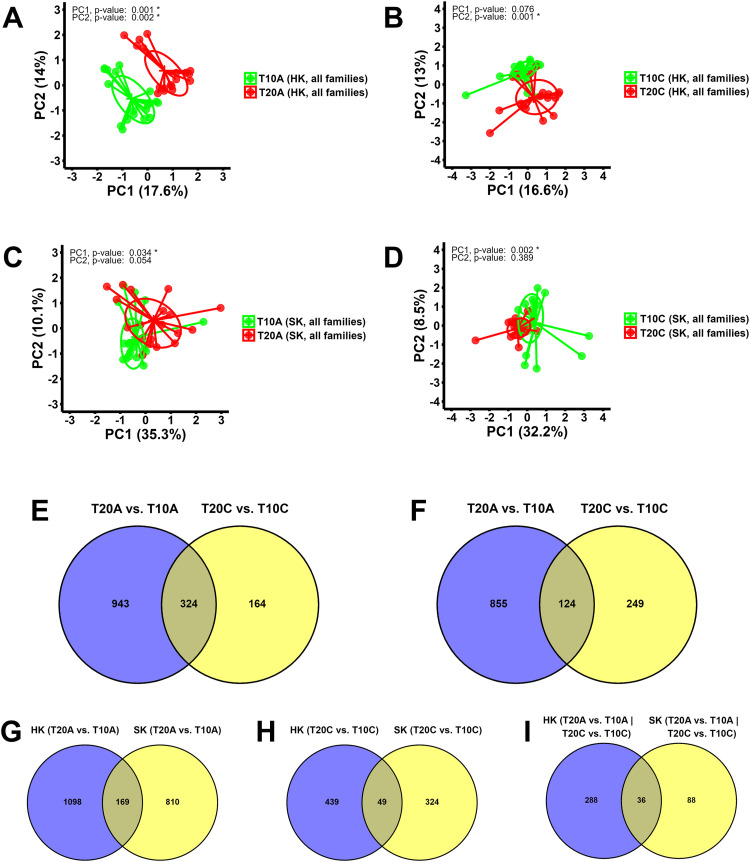
Principal Component Analysis (PCA) plots exhibiting the distribution of RNA-seq samples based on raw count data from all expressed genes and Venn diagrams representing head kidney and skin transcriptome responses of Atlantic salmon infested with adult or chalimus stages of sea lice under elevated (20 °C) versus normal (10 °C) temperature conditions. (**A-B**) PCA plots comparing head kidney samples from all families infested with adult (**A**) or chalimus (**B**) stages of sea lice at 20 °C vs. 10 °C. (**C-D**) PCA plots comparing skin samples from all families infested with adult (**C**) or chalimus (**D**) stages of sea lice at 20 °C vs. 10 °C. (**E-F**) Venn diagrams presenting the distribution of temperature-responsive differentially expressed genes (DEGs) identified in the head kidney (**E**) and skin (**F**) of families infested with adult or chalimus stages of lice. (**G-H**) Venn diagrams showing the distribution of temperature-responsive DEGs identified in both the head kidney and skin of families infested with adult (**G**) or chalimus (**H**) stages of lice. (**I**) Venn diagram showing the distribution of temperature-responsive DEGs identified in both the head kidney and skin based on interaction of shared DEGs between families infested with adult and those parasitized with chalimus stages of lice.

Following infestation with either adult or chalimus stages, temperature-based pairwise comparisons (20 °C vs. 10 °C) were conducted both within individual families (Supplementary Fig. 8, Supplementary Table 3; Supplementary Data Files 11–16) and across all families combined (Table 4; Supplementary Data Files 17–18). PCA plots generated using the expression data associated with comparisons within specific families (Supplementary Fig. 9) showed distinct segregation among samples related to different families based on temperature (T20A vs. T10A or T20C vs. T10C) in most cases in both the skin and head kidney (*p* < 0.05 for PC1 or PC2). As indicated in Fig. 2, PCAs related to the expression data of comparisons between all families resulted in distinct (T20A vs. T10A in the head kidney, *p* < 0.05 for PC1 and PC2) or moderate (T20C vs. T10C in the head kidney; T20A vs. T10A and T20C vs. T10C in the skin) separation of samples based on temperature (*p* < 0.05 for PC1 or PC2).

**Table 4:** Differentially expressed genes (DEGs) obtained based on pairwise comparisons of all salmon families parasitized with chalimus or adult stages of lice at two different temperature conditions for each tissue.

Pairwise comparisons	No. of DEGs in skin			No. of DEGs in head kidney			No. of DEGs shared between both tissues
	Up	Down	Total	Up	Down	Total	Total
T20A vs. T10A							
All families	556	423	979	775	492	1267	169
T20C vs. T10C							
All families	165	208	373	231	257	488	49
T20A vs. T10A | T20C vs. T10C							
Shared DEGs			124			324	36

**Fig. 3:**
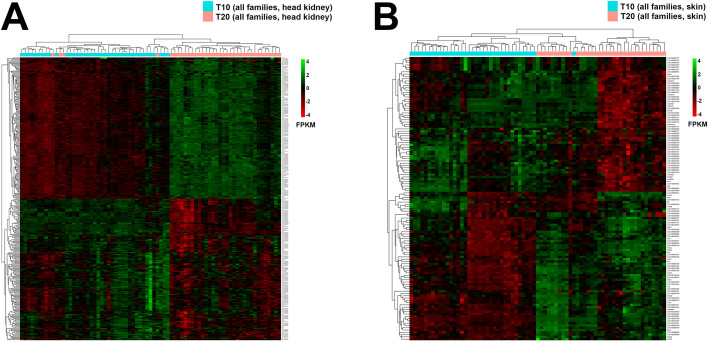
Hierarchical clustering of temperature-responsive differentially expressed genes (DEGs) shared between families parasitized with adult or chalimus stages of sea lice (T20A vs. T10A | T20C vs. T10C). (**A**) The left heatmap illustrates the hierarchical clustering of temperature-responsive DEGs (n=324) in head kidney tissue. (**B**) The right heatmap shows the hierarchical clustering of temperature-responsive DEGs (n=124) in skin tissue.

DEGs and GO/KEGG pathways obtained based on the combination of all families (Supplementary Data Files 17–18) were more comprehensive and overlapped, predominantly with those from within-family comparisons (Supplementary Data File 10, Supplementary Data Files 11–16), and therefore they are presented and discussed in this study. As summarized in Table 4, pairwise comparisons between all families infested with adult (T20A vs. T10A) and chalimus (T20C vs. T10C) stages at two different temperatures resulted in identification of 979 (556 up-regulated and 423 down-regulated) and 373 (165 up-regulated and 208 down-regulated) DEGs in the skin and 1267 (775 up-regulated and 492 down-regulated) and 488 (231 up-regulated and 257 down-regulated) DEGs in the head kidney, respectively. The interaction between these two comparisons (T20A vs. T10A | T20C vs. T10C) revealed 124 and 324 shared DEGs in the skin and head kidney, respectively (Fig. 2). A heatmap constructed based on the shared DEGs identified in the skin (n=124) and head kidney (n=324) indicated clustering of infested samples based on temperature (Fig. 3).

**Fig. 4:**
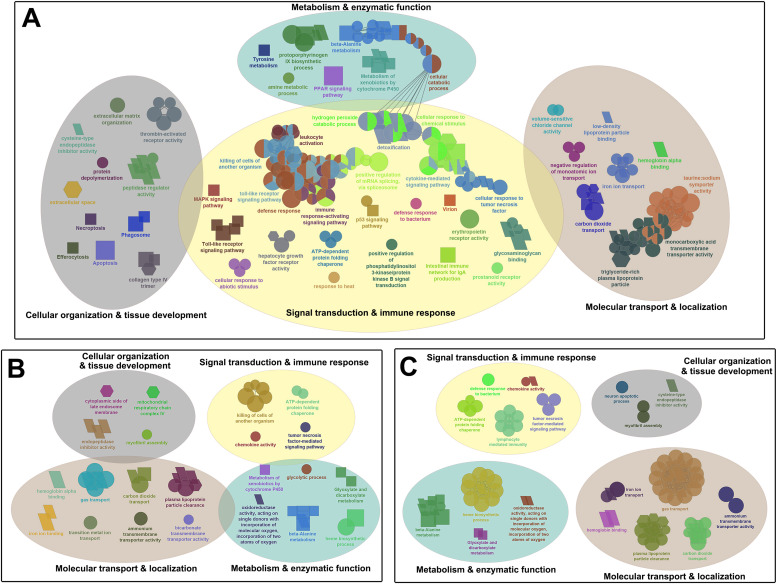
Functionally grouped network visualization of enriched GO terms and KEGG pathways based on different temperature conditions. (**A-B**) GO terms and KEGG pathways enriched based on DEGs identified in the head kidney of all families infested with adult (**A**) or chalimus (**B**) stages of sea lice under elevated (20 °C) versus normal (10 °C) temperature conditions. (**C**) GO terms and KEGG pathways enriched based on DEGs shared between the head kidney of families infested with adult or chalimus stages of sea lice at 20 °C vs. 10 °C. The leading GO terms and KEGG pathways were assigned into 4 functional themes including metabolism and enzymatic function (1), cellular organization and tissue development (2), signal transduction and immune response (3), and molecular transport and localization (4). The GO domains and KEGG pathways are represented as follows: GO biological process (ellipse), GO cellular component (hexagon), GO molecular function (parallelogram), KEGG pathways (rectangle).

**Fig. 5:**
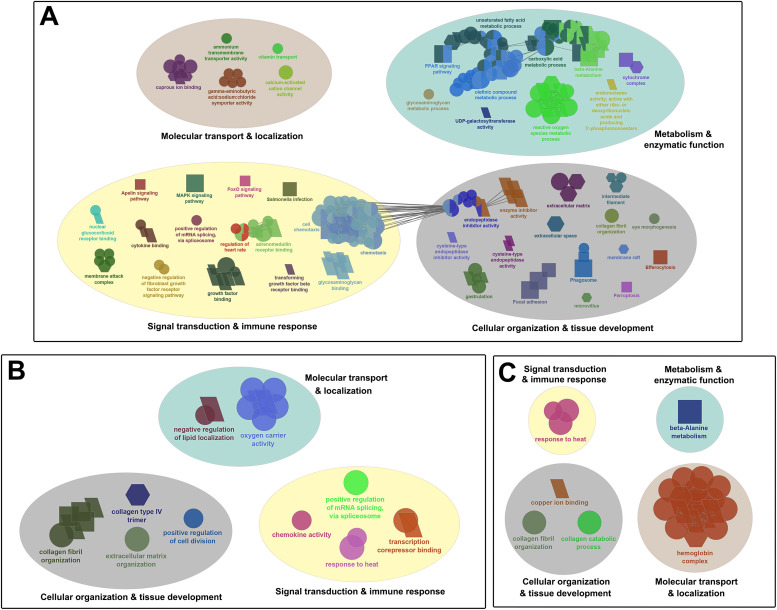
Functionally grouped network visualization of enriched GO terms and KEGG pathways based on different temperature conditions. (**A-B**) GO terms and KEGG pathways enriched based on DEGs identified in the skin of all families infested with adult (**A**) or chalimus (**B**) stages of sea lice under elevated (20 °C) versus normal (10 °C) temperature conditions. (**C**) GO terms and KEGG pathways enriched based on DEGs shared between the skin of families infested with adult or chalimus stages of sea lice at 20 °C vs. 10 °C. The leading GO terms and KEGG pathways were assigned into 4 functional themes including metabolism and enzymatic function (1), cellular organization and tissue development (2), signal transduction and immune response (3), and molecular transport and localization (4). The GO domains and KEGG pathways are represented as follows: GO biological process (ellipse), GO cellular component (hexagon), GO molecular function (parallelogram), KEGG pathways (rectangle).

In general, a higher number of leading/sub-leading GO terms were enriched in the head kidney and skin of fish infested with adult compared to those infested with chalimus stages of lice at 20 °C versus 10 °C (T20A vs. T10A > T20C vs. T10C). Likewise, the abundance (A), expression range (ER) and intensity (I; transcripts with log_2_FC ≥ ±3) of DEGs identified based on temperature differences were generally higher in both the head kidney and skin of adult-infested fish (A: 1267 and 979; ER: -4.69 to 6.53 and -4.29 to 7.06; I: 73 and 56, respectively) compared to those of chalimus-infested fish (A: 488 and 373; ER: -3.97 to 4.35 and -4.29 to 4.61; I: 9 and 11, respectively), suggesting greater transcriptional diversity in adult- versus chalimus-infested fish. The leading GO terms and KEGG pathways obtained based on DEGs identified in the head kidney and skin following temperature-based pairwise comparisons and their interactions (T20A vs. T10A, T20C vs. T10C, or T20A vs. T10A | T20C vs. T10C) were categorized into 4 functional themes as follows: (1) metabolism and enzymatic function, (2) cellular organization and tissue development, (3) signal transduction and immune response, (4) molecular transport and localization (Figs. 4 and 5).

Within the ‘metabolism and enzymatic function’ theme, enriched terms primarily reflected enhanced metabolic processes, including pathways associated with organic compound metabolism, energy production and lipid regulation (e.g., “glycolytic process” in the head kidney; “carboxylic acid metabolic process” in the skin; “PPAR signaling pathway” in both tissues). In addition, enrichment of pathways related to xenobiotic metabolism and oxidative stress responses indicates activation of detoxification mechanisms under elevated temperature and lice infestation. These responses were accompanied by enrichment of enzyme-related functions (e.g., “oxidoreductase activity” in the head kidney; “endonuclease activity” in the skin), suggesting altered catalytic activity under combined thermal and parasitic stress. The ‘cellular organization and tissue development’ theme was dominated by processes associated with tissue remodeling and structural reorganization, including extracellular matrix structure, cell-matrix interactions and proteolytic regulation (e.g., “extracellular matrix organization” and “cysteine-type endopeptidase inhibitor activity” in the head kidney and skin). Furthermore, enrichment of pathways related to programmed cell death (e.g., “apoptosis” and “necroptosis” in the head kidney; “ferroptosis” in the skin; “efferocytosis” in both tissues), together with processes linked to cellular proliferation, suggests tissue turnover and repair in response stress-inducing conditions.

The functional themes ‘signal transduction and immune response’ and ‘molecular transport and localization’ encompassed broader and more complex gene networks. Enriched pathways within the ‘signal transduction and immune response’ theme indicated activation of multiple immune and stress-related signaling cascades, including pathways associated with cytokine signaling, tumor necrosis factor (TNF) signaling, mitogen-activated protein kinases (MAPK) signaling, heat shock response and RNA processing. In the head kidney, *jun/ap1* together with genes from several key signaling families were differentially expressed, including the TNF superfamily (TNFSF) (*traf2b* [up], *traf2* [down], *cd40* [up], *fas* [down], *faslg* [up]), heat shock proteins (HSPs) (*hsp90aa1* [up], *hspa8* [up]), protein kinases (PKs) (*prkcb* [up], *jak1* [up]), MAPKs (*mapk11* [down], *mapk12a* [down], *map3k11* [up], *mapkapk2* [up], *mapk8ip2* [up]), Toll-like receptors (TLRs) (*tlr2* [up], *tlr7* [up], *tlr8* [up–down], *tlr12* [up]), growth factor ligands/receptors (*met* [up], *kdr* [up], *fgf10a* [up]), and members of the rat sarcoma virus (RAS) superfamily (*rac3* [up], *rasgrf2* [up], *rasgrp3* [up]). These genes were associated with over-represented pathways such as “MAPK signaling pathway”, “Toll-like receptor signaling pathway”, “NOD-like receptor signaling pathway” and “GnRH signaling pathway”. In the skin, enrichment of the “MAPK signaling pathway” was similarly supported by DEGs including *jun*/*ap1, dusp1* (up), *dusp4* (up), *vegfa* (up), *kdr* (up), *flt1* (up), *ddit3* (up), *gadd45a* (up), *flt3* (down), *map4k2* (down) and *map3k11* (down). Together, these patterns suggest a coordinated response involving modulation of cellular stress signaling and immune-inflammatory pathways, particularly under elevated temperature and adult-stage infestation.

In parallel, enrichment of terms related to the theme ‘molecular transport and localization’ reflected increased demands for substrate exchange, ion balance and gas transport, involving pathways associated with oxygen transport, iron metabolism, lipoprotein homeostasis and diverse transmembrane transport activities. Consistent with these patterns, several genes linked to molecular transport and cellular homeostasis, particularly members of the solute carrier (SLC) transporter family, were differentially expressed under elevated temperature. These included transporters associated with amino acids, ions, metabolites and neurotransmitters (up: *slc6a6, slc38a4, slc6a20, slc5a7*; down: *slc6a13, slc16a6, slc6a14, slc29a1, slc39a4, slc25a37, slc5a7, slc4a1, slc16a3b*), indicating modulation of substrate transport and metabolic regulation in the head kidney and/or skin. Collectively, these responses highlight the importance of maintaining physiological homeostasis and supporting metabolic and immune functions under environmental and pathogenic stress.

**Fig. 6:**
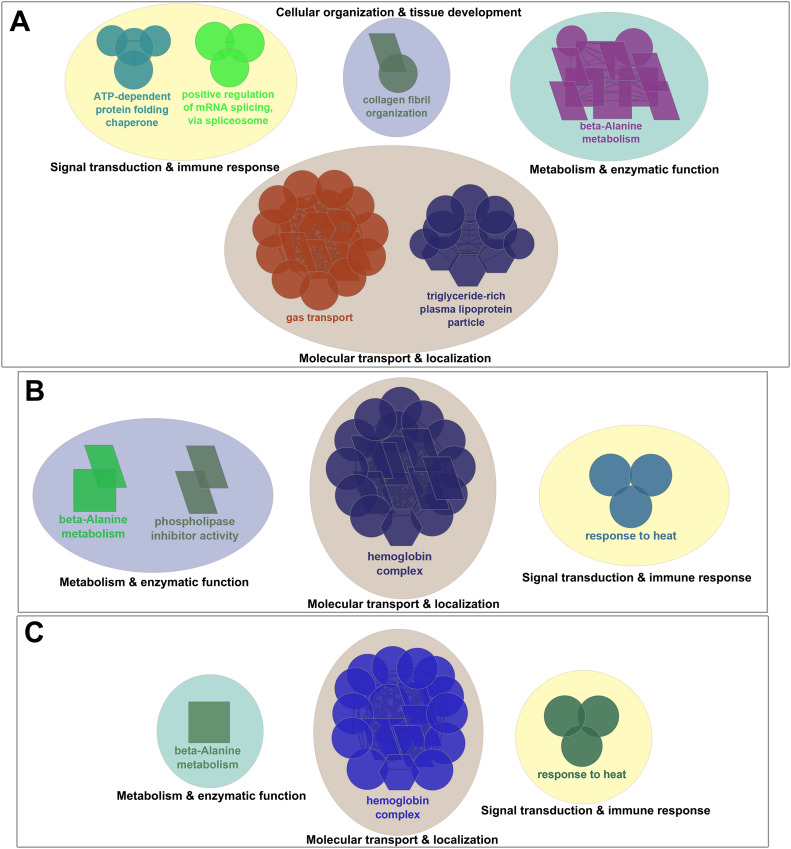
Functionally grouped network visualization of enriched GO terms and KEGG pathways based on different temperature conditions. (**A**) GO terms and KEGG pathways enriched based on DEGs shared in both the head kidney and skin of families infested with adult lice at 20 °C vs. 10 °C. (**B**) GO terms and KEGG pathways enriched based on DEGs shared in both the head kidney and skin of families infested with chalimus stages of sea lice at 20 °C vs. 10 °C. (**C**) GO terms and KEGG pathways enriched based on temperature-responsive DEGs identified in both the head kidney and skin of families infested with either adult or chalimus stages of sea lice. The leading GO terms and KEGG pathways were assigned into 4 functional themes including metabolism and enzymatic function (1), cellular organization and tissue development (2), signal transduction and immune response (3), and molecular transport and localization (4). The GO domains and KEGG pathways are represented as follows: GO biological process (ellipse), GO cellular component (hexagon), GO molecular function (parallelogram), KEGG pathways (rectangle).

When interactions between DEGs obtained in the head kidney and skin were investigated, there were 169 and 49 common DEGs between the head kidney and skin of salmon infested with adult (T20A vs. T10A) or chalimus (T20C vs. T10C) stages of lice, respectively (Fig. 2). As illustrated in Fig. 2, the interaction between groups subjected to infestation resulted in identification of 36 (34 characterized DEGs, see Table 5 for details) temperature-responsive DEGs shared in both tissues (T20A vs. T10A | T20C vs. T10C). The leading GO terms enriched in both head kidney and skin of fish parasitized with chalimus/adult forms of lice involved “beta-alanine metabolism”, “hemoglobin complex” and “response to heat” (Fig. 6). Temperature-associated GO terms and KEGG pathways enriched from DEGs shared across both tissues following infestation with either chalimus or adult lice stages are detailed in Supplementary Table 4, Supplementary Table 5 and Supplementary Data Files 19–21. The expression of several genes was consistently higher across both tissues, including those associated with stress response and protein folding (e.g., *hsp90aa1, serpinh1, hspa8, dnaja, serf2*), lipid metabolism (*apoc1*, LOC106577511) and immune signaling (*cxcl10, mbl, cleca*). In contrast, genes related to oxygen transport (*hba, hbb*), membrane transport (*slc16a6*) and cellular structure (*cfap44, tmem269*) showed lower expression levels in both tissues.

**Table 5:** Temperature-responsive differentially expressed genes (DEGs) identified in both head kidney and skin tissues of families (combined) infested by chalimus or adult stages of lice (T20A vs. T10A | T20C vs. T10C).

Gene ID	Gene symbol	Gene description	Function	Fold-change (log_2_FC)			
				HK (T20A vs. T10A)	HK (T20C vs. T10C)	SK (T20A vs. T10A)	SK (T20C vs. T10C)
LOC106581278	*serpinh1*	Serpin H1	Collagen biosynthesis and maturation	4.77	3.62	4.16	2.92
LOC106613072	*serpinh1*	Serpin H1		3.84	3.00	3.74	2.46
*hs90a*	*hsp90aa1*	Heat shock protein HSP 90-alpha	Protein folding regulation and stabilization	3.90	1.67	3.59	1.52
LOC106608136	*hsp90aa1*	Heat shock protein HSP 90-alpha		3.21	1.88	3.48	1.62
*hsc70*	*hspa8*	Heat shock cognate 70	Protein folding and apoptosis regulation	2.61	1.28	1.73	1.80
*dnaja*	*dnaja*	DnaJ heat shock protein family (Hsp40) member A	Protein folding regulation	2.38	1.37	2.75	2.48
LOC106584915	*ficd*	Protein adenylyltransferase FICD	Regulation of the UPR response	1.34	1.57	1.27	1.437
LOC100194731	—	Primary amine oxidase, liver isozyme	Amine metabolism, detoxification	4.82	2.68	3.40	1.70
LOC106577511	*apoc1*	Apolipoprotein C-I	Lipoprotein metabolism	4.18	3.65	3.46	2.44
*apoc1*	*apoc1*	Apolipoprotein C-I		2.76	2.15	-2.84	-1.90
*gadl1*	*gadl1*	Glutamate decarboxylase like 1	Beta-alanine synthesis and carnosine production	2.13	1.39	2.76	1.54
LOC106594092	*gadl1*	Acidic amino acid decarboxylase GADL1		2.09	1.40	2.96	1.25
LOC123724424	*plaat4*	Phospholipase A and acyltransferase 4	Phospholipid metabolism, keratinocyte differentiation and anti-pathogen defense	1.72	1.46	2.07	1.32
*prmt2*	*prmt2*	Protein arginine methyltransferase 2	Methylation, transcription regulation and alternative splicing	1.42	1.37	2.27	1.41
*cxl10*	*cxcl10*	C-X-C motif chemokine 10	Immune response modulation	2.78	1.82	4.85	2.29
LOC100136446	*cleca*	C type lectin receptor A	Pathogen recognition	2.37	1.32	1.84	1.47
LOC106601909	*mbl*	Mannose-binding protein	Pathogen recognition	1.51	1.28	3.04	1.43
LOC106604762	*tspan7*	Tetraspanin-7	Cell Adhesion/signaling and neuronal development	2.74	2.02	2.73	2.57
LOC106571140	*papln*	Papilin	ECM organization	2.65	2.41	-1.44	-1.27
LOC106578921	*snca*	Alpha-synuclein	Synaptic vesicle regulation	2.39	1.49	3.56	1.75
LOC106591183	*serf2*	Small EDRK-rich factor 2	Protein destabilization	1.58	1.35	1.86	1.59
LOC106561282	*cfap44*	Cilia- and flagella-associated protein 44	Microtubule cytoskeleton organization	-1.28	-1.31	-1.99	-1.27
LOC106567151	*tmem269*	Transmembrane protein 269	Signal transduction and protein stability	-1.53	-1.28	-2.74	-2.13
LOC106593002	*slc16a6*	Monocarboxylate transporter 7	Monocarboxylic acid transmembrane transporter activity	-1.62	-1.42	-2.03	-2.08
LOC106594081	*slc5a7*	High-affinity choline transporter 1	Choline uptake	-2.56	-1.33	1.74	1.53
LOC106592262	*cyp4f3*	Cytochrome P450 4F3	Inflammation control	-1.98	-1.33	-3.24	-1.73
*hmgb3*	*hmgb3*	High mobility group protein B3	DNA binding	-4.35	-1.62	-3.87	-3.63
*tfa*	*tfa*	Transferrin-a	Iron transport	1.60	1.65	-1.38	-2.90
LOC106601072	*hba1*	Hemoglobin subunit alpha	Oxygen transport	-2.62	-2.01	-1.86	-1.37
LOC106601077	*hba1*	Hemoglobin subunit alpha		-2.91	-1.91	-1.83	-1.44
LOC106607373	*hba1*	Hemoglobin subunit alpha		-3.33	-1.51	-2.29	-1.55
LOC106607372	*hbb*	Hemoglobin subunit beta	Oxygen transport	-2.71	-1.29	-2.13	-1.28
LOC106601074	*hbb1*	Hemoglobin subunit beta-1	Oxygen transport	-2.90	-1.78	-2.06	-1.38
LOC106607371	*hbb1*	Hemoglobin subunit beta-1		-3.15	-1.53	-2.85	-1.45

### qPCR data analysis

#### Differential gene expression between families

qPCR data were analyzed across infestation stages and temperature conditions (T10A, T10C, T20A, and T20C) in both tissues (Supplementary Figs. 10–17). Differential expression among families was limited and observed only in head kidney tissue, where *clec4* (T10A, T10C, T20A), *txnb* (T10A), and *ncf2* (T10C) showed significant differences. No significant expression differences were detected in skin tissue. RNA-seq analysis partially supported these findings, identifying differential expression of *clec4* family members, although no differences were observed between F419 and other families. Both approaches consistently showed minimal expression of *cd83, col1a1, mmp9, saa5* and *timp2* in head kidney tissue. In contrast, RNA-seq indicated variable expression of *clec4, mmp9, saa5* and *timp2* in skin tissue, which was not detected by qPCR. These discrepancies likely reflect differences in sample size, normalization methods (TMM vs. ΔΔCt), variation in sampled individuals (particularly for skin tissue) and targeting of different gene paralogs in qPCR assays (particularly for *clec4*).

#### Effect of temperature on gene expression

**Fig. 7:**
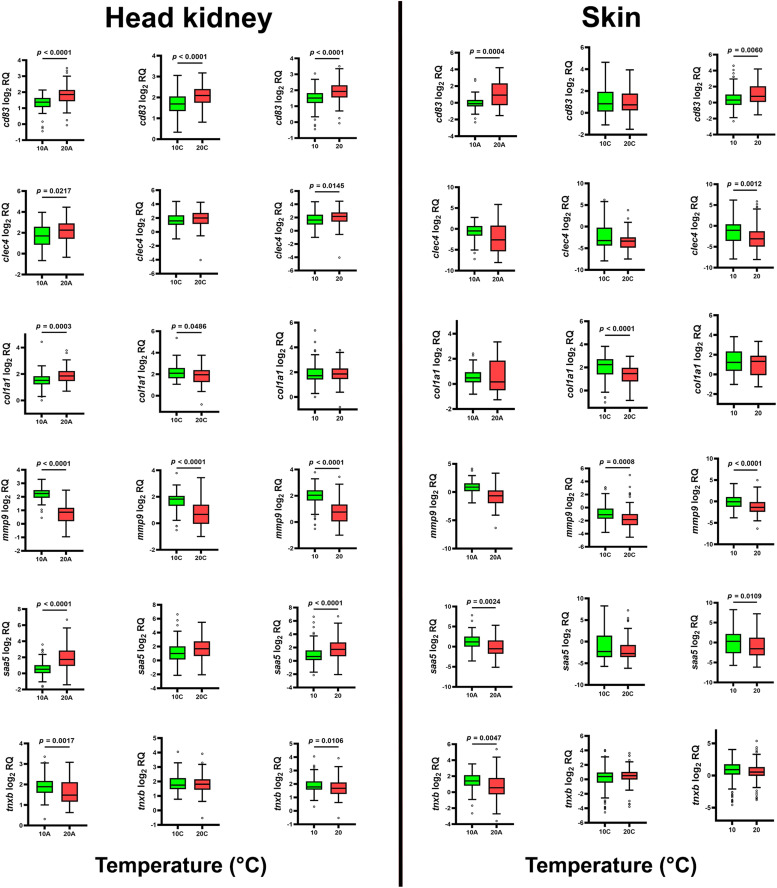
qPCR results for *cd83, clec4, col1a1, mmp9, saa5* and *txnb* in the head kidney and skin of salmon families infested under elevated (20 °C) and normal (10 °C) temperature conditions. Data are presented as box plots with median values and Tukey whiskers. Significant differences (*p* < 0.05) in gene expression responses between families under the two temperature conditions are indicated by asterisks. For each gene, expression responses to temperature were analyzed in salmon infested with adult lice (left plot), chalimus lice (middle plot) and across all infested salmon regardless of stage (right plot).

Temperature effects on gene expression were independent of family; therefore, all fish were pooled by temperature for analysis (Supplementary Fig. 18). qPCR comparisons were conducted between 20 °C and 10 °C under chalimus (T20C vs. T10C), adult (T20A vs. T10A) and combined infestation conditions (chalimus + adult; T20 vs. T10) (Fig. 7). A significant positive correlation (*p* < 0.01) was observed between qPCR and RNA-seq datasets (Supplementary Fig. 19). qPCR identified differential expression of *cd83, clec4, col1a1, mmp9, saa5* and *txnb* in both tissues. *cd83* was consistently up-regulated at elevated temperature across both methods. *clec4* showed tissue-specific patterns, with up-regulation in the head kidney and down-regulation in the skin, supported by RNA-seq evidence of paralog-specific regulation (*clec4m* was down-regulated and *clec4e* was mainly up-regulated in the head kidney, while *clec4e* and *clec4f* were up-regulated in the skin). *col1a1* exhibited stage-dependent responses in the head kidney and consistent down-regulation in the skin at elevated temperature. RNA-seq analysis, based on within-family or combined-family comparisons, supported an overall down-regulation of *col1a1* (LOC106610502) in both tissues.

*mmp9* expression was reduced in both tissues at 20 °C, consistent between RNA-seq and qPCR (Fig. 7). In contrast, *saa5* showed tissue-specific responses, with qPCR indicating up-regulation in the head kidney but down-regulation in the skin of adult-infested fish (*p* < 0.05), while RNA-seq revealed up-regulation in both tissues during adult infestation and down-regulation in the skin during chalimus infestation. *txnb* was down-regulated in both tissues of adult-infested salmon based on qPCR, which was supported by RNA-seq in the head kidney but not in the skin, where RNA-seq showed up-regulation. No significant expression changes were detected for *ncf2* and *timp2* in either dataset (Supplementary Fig. 20). These discrepancies may be attributed to differences in sample size, given that a larger number of families and individuals were included in the qPCR assays, as well as the combined effects of temperature and infestation stage, suggesting context-dependent gene expression. Interactive effects between these variables may lead to expression patterns that are detectable in the RNA-seq dataset but less evident under the specific conditions assessed by qPCR. Overall, RNA-seq provides a broader overview, and discordant results should be interpreted cautiously.

## Discussion

### Family-based transcriptomic responses to sea lice infestation

The salmon families included in the RNA-seq analyses, which exhibited different MLDs, were not pre-classified as resistant or susceptible; therefore, the differentially expressed genes identified in this study should be considered as candidate markers associated with variation in lice densities, rather than definitive biomarkers of resistance or susceptibility. Future studies that combine transcriptomics with families of known phenotypes or genetic association analyses will be needed to confirm their role in resistance/susceptibility to sea lice infestation. High variability in lice density was observed within certain families (e.g., F175 at 20 °C). Although our transcriptomic comparisons were performed between multiple families, this variability may introduce additional noise into the identification of resistance-associated genes. To mitigate this effect, candidate genes were prioritized based on reproducibility across different comparisons, functional relevance and consistent expression differences between families with contrasting lice densities. Nonetheless, we acknowledge that family-level variation remains an important factor to consider when interpreting the results. Despite the differences between lice density in infested families, the transcriptional changes and gene expression profiles identified in the skin and head kidney were also highly variable among families. Of the selected DEGs related to family-based comparisons (Table 3), *kbas, nlrp1, nlrc3, nlrp12, mmp13, muc2, sntx-b* and *trim16* represented several paralogs with mostly identical or reverse expression patterns in the skin and/or head kidney.

Although lice density did not differ significantly among salmon families included in RNA-seq in most cases, those with relatively lower lice densities (F361 and F265) exhibited the most pronounced transcriptional differences. The expression profiles of several genes differed in F361 and F265 compared with other families. Notably, F361 showed the strongest modulation, consistent with lice density patterns observed at both 10 °C and 20 °C for F361 and at 10 °C for F265. These responses included the modulation of multiple immune-related genes that may contribute to reduced susceptibility to sea lice infestation (see discussion below). It is also important to consider that the lack of statistical significance in lice burden does not necessarily preclude biologically meaningful differences among families [24]. In sea lice infestations, parasite distribution on the host is spatially heterogeneous; consequently, sampled tissues may not always coincide with parasite attachment sites, introducing variability and potentially reducing the sensitivity of statistical comparisons among families.

Among immune-related genes, the expression levels of *cd209, cd209c, cd209e,clec4m* [*cd209l*] and *clec4e* [*mincle*] were higher in F361 compared to other families in both the head kidney and skin. On the other hand, *cd209d* was among genes with lowest expression in F361 compared to F175 and F419. In addition, *cd244* displayed lower expression levels in F361 relative to other families. Increased transcript level of *btn2a1* which serves as a ligand for *cd209* was also detected in the skin of F361 compared to other families [41]. *cd209* (also known as *dc-sign*) which is primarily expressed on the surface of dendritic cells is crucial for initiating immune responses against pathogens such as sea lice by recognizing and binding to their specific sugar structures [8,12]. Likewise, the induced expression of *mincle* is associated with resistance against sea lice in coho [12], pink [40] and Atlantic salmon [9]. Moreover, the expression of *clec10a* (in the skin) and *ctsc* (in the head kidney and skin) was found to be higher in F361 compared to other families. The *ctsc* gene (cathepsin C) is a lysosomal aminopeptidase essential for the activation of multiple serine proteases in immune cells, enhancing their ability to fight pathogens [42]. *clec10a* (*mgl*) which is expressed mainly on dendritic cells recognizes *N*-acetylgalactosamine (GalNAc) residues and promotes the production of *il10* that regulates immune responses [43]. Besides, enhanced expression of *mrc1* and *lamp3* (*dc-lamp* or *cd208*) was noticed in the skin and/or head kidney of F361 compared to other families. *mrc1* is responsible for recognizing carbohydrate/chitin structures on parasites [44], and its association with resistance to sea lice infestation has been previously demonstrated in coho and Atlantic salmon [9,12]. *lamp3* is expressed on the surface of immune cells like dendritic cells and involved in MHC class II antigen presentation and immune activation [45]. *clec3ba, siglec6* and *colec12* were also expressed at higher level in the skin of F361, although these changes were identified in few comparisons (T10A, F361 vs. F175, F419 and F265). *siglec6* is primarily expressed by mast cells and limits excessive inflammatory responses [46].

*psmb9a* was more abundantly expressed in the skin and head kidney of F361 compared to other families. This gene encodes a subunit of the immunoproteasome that is essential for processing antigens that are presented on MHC class I molecules for recognition by CD8^+^ T cells [47]. Enhanced transcript level of *nlrp1* was observed in the skin and head kidney of F361 and F265 compared to the other families infested with adult/chalimus stages of lice at 10 °C and/or 20 °C. *nlrp1* is a key inflammasome sensor that is activated by pathogen-associated molecular patterns (PAMPs) and contributes to the recruitment of immune cells to the site of infection for clearance of pathogens [48]. The expression of *rock2* and *il1b*, on the other hand, was found to be reduced in the skin of F361 and F265 compared to other families (specially F419). Downregulation of *rock2* has been associated with restoration of immune balance in pro-inflammatory conditions [49]. The expression level of some key components related to innate immune system (*nkl, pglyrp2, prf1.3* in the head kidney, *psap, h2-q9* and *c1qb* in the head kidney and skin) was higher in F361 relative to other families. NK-Lysin and perforin are predominantly produced by cytotoxic T lymphocytes and natural killer (NK) cells and are involved in defense against invading pathogens [50,51]. *h2-q9* (encoding Qa2 protein) is a mouse-associated non-classical MHC class Ib molecule that has been shown to present peptides to CD8^⁺^ T cells [52,53]. Interestingly, Qa2 was demonstrated to participate in resistance to murine cysticercosis caused by *Taenia crassiceps* [54]. As an integral part of the C1q complex, *c1qb* gene plays a pivotal role in the classical pathway of the complement cascade by the recognition/elimination of foreign particles and clearance of immune complexes. C1q has been also suggested to regulate macrophage polarization toward an anti-inflammatory phenotype and inhibit inflammasome activation [55]. In addition, lowered expression of *palm3* was detected in the skin and head kidney of F265 and F361 compared to F175 and F419. Overexpression of *palm3* is associated with exacerbated inflammatory response, whereas its downregulation can suppress the induced inflammation [56]. Unlike perforin (*prf1.3*), the expression of genes encoding aerolysin-like protein (down in the skin) and stonustoxin subunit beta (*sntx-b*, down in the head kidney and up-down in the skin) was mainly reduced in F361 compared to other families. Aerolysin and stonustoxin may serve as a defence system against parasites such as sea lice, but their exact roles remain to be elucidated [57,58]. *kbas* and *ighd* exhibited higher transcription in the skin of F361 relative to other families, suggesting their possible engagement in immune responses against *L. salmonis*.

The expression of genes related to keratin and collagen families (e.g., *krt13, krt8.2.l* and *col6a2*) was predominantly higher in F361 than other families, likely providing a stronger barrier against mechanical stress and parasite invasion [59]. Decreased expression of *mmp9, mmp13* and *timp2* in F361 could potentially be indicative of a more controlled tissue damage and promoted healing process in this family. Further, *tgm1, tgm2* and *tgm3* showed reduced transcript levels in the skin F361 and F265, which may suggest minimal tissue damage and less stressful conditions in these families [60]. Some heat shock proteins (HSPs) exhibited reduced expression in F361 compared to other families (*hspa14* and *hspd1* in the skin; *hspa8* in the head kidney and skin), which based on lower parasite load observed in F361 may indicate less cellular damage or a more efficient stress tolerance mechanism adopted by this family. Several members of the myosin and troponin family were modulated (e.g., *tpm2, tnnc2, tnni1, myha, mylk3* and *myl3*) in response to infestation with the chalimus stage of sea lice, and transcription of most of which was found to be lower at 10 °C but higher at 20 °C in F361 and F265 compared to other families. Modulated expression of biomarkers associated with muscle function/stress could be attributed to increased physical activities of Atlantic salmon following infestation with chalimus stages of sea lice [9]. A variety of hemoglobin (e.g., *hba1, hba4, hbb, hbb1* and *hebp2*; mostly up in both tissues) and ferritin (*ftm*, up-down in both tissues) subunits represented varied expression in F361 compared to other families. These genes were also present in several enriched GO terms including “cellular detoxification”, “antioxidant activity”, “sequestering of iron ion” and/or “oxygen transport”, suggesting their involvement in iron homeostasis and defense against sea lice [9,11,13,35].

### Transcriptomic changes associated with incremental temperature

The general response to high temperature stress occurs at three levels: primary (release of catecholamines and corticosteroids), secondary (metabolic changes and energy mobilization) and tertiary (inhibition of growth, reproduction and immunity) [18,19]. The transcriptional changes associated with elevated temperature have been previously studied in healthy/challenged salmonids [[61], [62], [63]]. In this study, the transcriptome responses of Atlantic salmon parasitized with chalimus or adult stages of sea lice were compared between standard (10 °C) and increased (20 °C) rearing temperatures. Here we discuss more abundant and/or significant genes that were predominantly expressed across both within- and combined-family’s comparisons.

Thermal stress disrupts the stability and function of proteins, leading to the production of HSPs which prevent protein denaturation/misfolding and maintain cellular functions [64]. The transcript levels of a number of HSPs (*serpinh1* [*hsp47*], *hsp90aa1, hspa8, dnaja* and *hsp90b1*) were enhanced in the head kidney and skin of salmon infested with chalimus or adult stages of lice at high temperature. These DEGs were also involved in GO terms associated with heat shock response (“response to heat”, “response to temperature stimulus” and “ATP-dependent protein folding chaperone”). *serpinh1* and *hsp90aa1* have been previously suggested as reliable markers of thermal stress in Atlantic salmon [63]. *serpinh1* is a collagen-specific chaperone in the endoplasmic reticulum that is implicated in proper folding and assembly of procollagen [65,66], whereas *hsp90aa1* is a molecular chaperone that plays a significant role in stress adaptation, protein homeostasis and cellular signaling under heat stress conditions [61]. Our findings suggest that *hspa8* and *dnaja* may also be considered as potential markers responsive to elevated temperature in Atlantic salmon, as they were up-regulated in both chalimus- and adult-infested salmon exposed to the higher temperature. The heat-induced up-regulation of *hspa8* and/or *dnaja* has been previously reported in other fish species including rainbow trout [[67], [68], [69]], channel catfish [70], Nile tilapia [71] and Atlantic sturgeon [72]. *atf6* and *atf3* were more abundantly expressed in the head kidney of adult-infested salmon maintained at the higher temperature (20 °C). *atf6* is a major regulator of the endoplasmic reticulum stress response that functions as a transcription factor in response to heat-induced protein misfolding. Upon cleavage in the Golgi apparatus, the active form of *atf6* induces genes involved in protein folding (e.g., chaperones like HSPs) and oxidative stress defense [73]. *atf3* is a stress-induced transcription factor that responds to heat, inflammation and oxidative stress [74]. The transcript level of *cirbp* and *fkbp10* decreased in salmon exposed to high temperature, consistent with previous findings in several salmonid species [61,63]. The cold-inducible RNA-binding protein (*cirbp*) gene encodes a stress-responsive protein involved in inflammation, cell proliferation, and apoptosis [75], while *fkbp10* gene codes for an endoplasmic reticulum-localized peptidyl-prolyl isomerase (PPIase) essential for proper folding and stabilization of collagen [76].

Some genes associated with the serine/arginine-rich splicing factors family (up: *srsf2* and *srsf6*; down: *srsf2b*), which play pivotal roles in regulating pre-mRNA splicing and mRNA metabolism, were modulated in the head kidney and skin of Atlantic salmon in response to increased temperature. Two paralogs annotated as elongation factor 2 (*eef2*) were expressed in the head kidney and skin of thermally exposed fish (20 °C), exhibiting opposite expression patterns. *eef2* encodes for a protein that promotes the GTP-dependent translocation of the ribosomes during protein synthesis [77]. *srsf2* and *eef2* have been previously suggested as robust biomarkers of chronic thermal stress in other salmonid species [61]. *tuba1a* (down in the head kidney; up in the skin), *tuba4a* (down in the head kidney and skin) and *tubb4b* (down in the head kidney; up-down in the skin) showed differential expression at higher temperature. These genes were also present in GO terms “gap junction” and “phagosome” in fish infested by adults at 20 °C. As components of microtubules, *tuba1a, tuba4a* and *tubb4b* are critical for maintaining cytoskeletal integrity, intracellular transport and cell division [78]. Different paralogs of *tuba1a* located on the chromosomes 11 and 16 exhibited opposite expression patterns in other salmonid species [61]. These results are congruent with earlier findings, suggesting that certain tubulins may serve as potential biomarkers of thermal stress in salmonids. *hmgb3* (up-down paralogs) and *hmgb3a* (down) were differentially expressed in the head kidney and skin of fish held at 20 °C [61]. High mobility group box (HMGB) proteins were found to be modulated in Atlantic salmon exposed to high temperature [79,80]. HMGB proteins are involved in cellular processes associated with DNA and can also act as cytokines during inflammation, infection and injury [81]. Up-regulated expression of *rgs5* was observed in the head kidney and skin of fish held at 20 °C. *rgs5* is a hypoxia-responsive protein involved in vascular function/remodeling and endothelial apoptosis via the p38 signaling pathway [82,83]. Higher expression of *prmt2* and *hnrnpc*, and lower expression of *dhx32, rps23, nmt1b* and *h1* (histone H1) were identified in the head kidney of salmon exposed to high temperature. These genes play essential roles in RNA processing, protein synthesis/stability and/or chromatin regulation, and their altered expression is likely associated with cellular adaptation to thermal stress [70,84].

Several biomarkers associated with oxidative stress/redox homeostasis were found to be modulated in salmon exposed to high temperature. For example, representatives of genes belonging to glutathione-S-transferase (GST) family had varied expression in fish infested at 20 °C (up: *gsta3* in the head kidney and skin; down: *mgst1* in the head kidney, *lancl1* in the head kidney and skin). *gsta3* is a GST α-class involved in cellular detoxification via conjugation of reduced glutathione to hydrophobic electrophiles [85], and *lancl1* gene codes for a GST enzyme that protects cells from oxidative stress by conjugating glutathione to xenobiotic substrates [86]. *mgst1* represents both glutathione peroxidase (GPx) and GST activities, thus catalyzing the reduction of lipid hydroperoxides and the conjugation of reduced glutathione to electrophiles [87]. These three genes were also involved in enriched GO terms “glutathione metabolism” and “glutathione transferase activity”, indicating their role in antioxidant defense of chalimus- and/or adult-infested salmon exposed to the higher temperature. The transcript level of *cdo1* (in the head kidney and skin) and *msrb2* (in the head kidney) was down-regulated in fish held at 20 °C. *msrb2* encodes an enzyme that plays a crucial role in protecting cells from oxidative damage by reducing oxidized methionine residues in proteins, thereby maintaining protein integrity and cell survival [88]. On the other hand, *cdo1* catalyzes the irreversible conversion of cysteine to cysteine sulfinic acid, indirectly influencing glutathione synthesis and potentially contributing to oxidative stress [89]. The exposure of fish to an elevated temperature resulted in down-regulation of *cat* (in the head kidney), *cbr1* (in the head kidney) and *bco2a* (in the skin). In addition, *txn* was down-regulated in the head kidney but up-regulated in the skin of fish infested by adult lice at 20 °C. Thioredoxin (*txn*) and catalase (*cat*) play crucial roles in maintaining redox balance, and their lowered expression in the head kidney could result in increased protein damage and apoptosis [63]. Loss or decreased expression of *bco2* has been associated with mitochondrial oxidative stress in mammals [90]. *cbr1* helps detoxify a variety of reactive carbonyl species, thus protecting cells from oxidative damage [91]. These findings collectively suggest that the antioxidant machinery was impacted in lice-infested salmon in response to thermal stress.

The SLC family comprises membrane-bound transporters that essentially act as gatekeepers to maintain cellular homeostasis [92]. A variety of genes related to this family were modulated in salmon exposed to the elevated temperature. In addition, some genes encoding members of the ATP-binding cassette (ABC) family (up: *abca12, abcb8*; down: *abcd3, abcd4, abcb10*) showed varied expressions in the head kidney of fish maintained at 20 °C. ABC transporters play an important role in maintaining cellular homeostasis under normal and stressful conditions by regulating multiple cellular processes including lipid transport, detoxification, ion balance and nutrient uptake [93]. The genes related to SLC/ABC transporters and/or apolipoproteins (*apoob, apoc1, apoeb*) were the major components of the GO terms “lipid transport”, “carboxylic acid transport”, “inorganic anion transmembrane transport”, “symporter activity” and “amino acid transmembrane transporter activity”. *sgk1* (up in the head kidney and skin of adult-infested salmon held at 20 °C) plays an important role in stress response by regulating a wide variety of cellular processes including ion channel activity, membrane transporter activity and cell growth/survival. Thermal stress-induced up-regulation of *sgk1* has been previously reported in the longjaw mudsucker (*Gillichthys mirabilis*) and the starry flounder (*Platichthys stellatus*) [94,95]. The expression of *atp1b1a* increased in the head kidney and skin of adult-infested salmon maintained at 20 °C. This gene codes for a subunit of the Na^+^/K^+^ ATPase protein pump, which plays a crucial role in osmotic regulation, ion balance and cellular signaling [96]. In contrast, *tmem269* represented lower expression in the head kidney and skin of salmon infested at 20 °C. *tmem269* encodes a poorly characterized membrane-associated protein that has been suggested to be involved in signal transduction, protein stability, and trafficking proteins to the membrane [97].

The modulation of *jun*/*ap1* under elevated temperature and lice infestation is consistent with its central role in regulating cellular stress responses, including the unfolded protein response (UPR) and activator protein-1 (AP-1)-mediated signaling pathways [98]. As a critical transcriptional regulator, *jun*/*ap1* integrates multiple upstream signals and modulates downstream processes involved in apoptosis, cell survival and inflammatory responses. Its temperature-associated expression pattern, also reported in other fish species [99,100], suggests an important role in mediating cellular adaptation to thermal stress. These responses may reflect a broader regulatory mechanism through which stress signaling pathways are engaged to maintain cellular function and stability under combined thermal and parasitic challenges [101,102].

Acute/chronic thermal stress has been shown to impact the immune inflammatory responses in fish [63,101]. Consistent with this, the expression of genes associated with inflammation and immune function was modulated under elevated temperature, as reflected by higher transcript levels of *camp, hamp, apoc1, c7b, c1ql2, c1ql4, saa5, ccl4, cxcl10, relt* and *mmp9* (in the head kidney and skin), as well as *lyz, prf1*, and *il6* (in the head kidney), and lower transcript levels of *litaf* (in the head kidney and skin) and *epx* (in the head kidney). GO terms represented mainly by up-regulated DEGs (“cell chemotaxis”, “innate immune response”, “adaptive immune response”, “positive regulation of response to stimulus”) were also enriched in the head kidney and/or skin. In addition, the enriched GO terms “enzyme inhibitor activity” and “cysteine-type endopeptidase inhibitor activity” included several DEGs with enzyme/protein inhibitor activities (e.g., *salarin, serpinh1, serpinh1a, serpine1, tfpi2, lcn, angptl4* [up in the head kidney and skin]; *cst* [up in the head kidney]; *timp2* [up-down in the skin]). Upregulation of protease inhibitors can promote cell survival by maintaining cellular proteostasis and reducing stress-induced apoptosis [103]. A diverse range of genes involved in metabolism of proteins (e.g., *cdo1* [up], *asrgl1* [up], *gpt2* [down], *gad1* [down], *gadl1* [up-down]), lipids (e.g., *plin2* [up], *lpl* [up], *angptl4* [up], *apoeb* [up in the head kidney; down in the skin]) and other organic/inorganic compounds (e.g., *cyp4f3* [down], *abat* [down], *gad1* [down]) were modulated in the head kidney and skin of salmon maintained at 20 °C. These DEGs were involved in several GO terms associated with metabolic processes such as “PPAR signaling pathway”, “amino acid catabolic process”, “organic acid biosynthetic process” and/or “small molecule catabolic process”. A number of genes involved in gas transport, heme biosynthesis, iron homeostasis and/or porphyrin metabolism (e.g., *hba1, hba4, hbb, hbb1* and *ca1* [in the head kidney and skin]; *cahz, alad, fech, uros, urod, hmbsa, ppox, hmbs* and *slc4a1* [in the head kidney]) were down-regulated following the exposure of fish to elevated temperature. These markers were involved in GO terms such as “oxygen carrier activity”, “bicarbonate transport”, “cellular detoxification” and “porphyrin metabolism”. The downregulation of these genes suggests that oxygen-binding efficiency, acid-base homeostasis, hemoglobin function and red blood cell stability were impacted in response to the higher temperature.

## Conclusion

Our findings suggest significant differences in molecular mechanisms across salmon families in response to sea lice. These findings underscore the importance of family-based transcriptomic analyses in identifying potential molecular biomarkers associated with sea lice resistance and susceptibility. Importantly, certain families (e.g., F361) exhibited more effective responses to sea lice infestation; however, these patterns may reflect parasite attachment dynamics shaped by host-parasite interactions, potentially influenced by differences in initial infestation and host behavioural traits, rather than intrinsic resistance alone. The family-based transcriptomic changes were observed in both the skin and head kidney, suggesting the complex localized and systemic impact of sea lice parasitism. Notably, several dendritic cell-associated receptors were differentially expressed between families with varying lice density, suggesting their pivotal role in triggering and regulating the immune response to sea lice infestation. This research revealed the profound transcriptomic alterations induced by elevated temperatures, with significant modulation of multiple genes and biological pathways involved in heat shock response, protein metabolism, immune system, and stress response. These changes suggest that thermal stress may exacerbate the overall impact of the parasite on salmon health, particularly over the long term. Although RNA-seq provides a broader and more precise overview of the transcriptomic responses than microarray technologies, the presence of a considerable number of uncharacterized genes (6–21 % per pairwise comparison in this study) in the current Atlantic salmon genome assembly poses a limitation in identifying other potential biomarkers associated with diseases/stressors in this species. Given the incomplete annotation and functional characterization of the Atlantic salmon genome, some gene assignments should be interpreted with caution. The biomarkers identified in this study provide deeper insights into the molecular mechanisms underlying the salmon immune responses to sea lice infestations across different thermal profiles. It should be noted that the expression differences observed in this study may reflect underlying genetic variation in other regions of the genome, such as regulatory loci or trans-acting factors. A comprehensive understanding of the genetic basis of resistance/susceptibility will therefore require complementary genomic analyses, including SNP genotyping, GWAS, TWAS or eQTL approaches. Overall, the candidate genes and putative biological pathways can be used as a reference for future studies focusing on development of new prevention strategies.

## Data availability

The sequence data supporting the findings of this study are available in the NCBI’s Sequence Read Archive (SRA) database under BioProject accession number PRJNA1256531 (http://www.ncbi.nlm.nih.gov/bioproject/1256531). Further information is available upon request from the corresponding authors.

## CRediT authorship contribution statement

**Reza Ghanei-Motlagh:** Writing – review & editing, Writing – original draft, Visualization, Formal analysis, Data curation. **Wenlong Cai:** Methodology, Formal analysis. **Jordan D. Poley:** Methodology, Formal analysis. **Yuxuan Zhang:** Methodology. **Shona K. Whyte:** Writing – review & editing, Project administration, Methodology. **Amber F. Garber:** Writing – review & editing, Supervision, Methodology, Funding acquisition, Conceptualization. **Mark D. Fast:** Writing – review & editing, Supervision, Methodology, Formal analysis, Conceptualization.

## Declaration of competing interest

The authors declare that they have no known competing financial interests or personal relationships that could have appeared to influence the work reported in this paper.

## Appendix Supplementary materials

Image, application 1

Image, application 2

Image, application 3

Image, application 4

Image, application 5

Image, application 6

Image, application 7

Image, application 8

Image, application 9

Image, application 10

Image, application 11

Image, application 12

Image, application 13

Image, application 14

Image, application 15

Image, application 16

Image, application 17

Image, application 18

Image, application 19

Image, application 20

Image, application 21

Image, application 22
