# Supplementary material for: Family-based transcriptomic profiling of Atlantic salmon skin and head kidney in response to Lepeophtheirus salmonis infestation under physiological and elevated temperatures
Source: Comp Immunol Rep. 2026 Apr 22;10:200285. doi: 10.1016/j.cirep.2026.200285 (PMC13142024; doi:10.1016/j.cirep.2026.200285)
Supplement: Supplementary file 1 [file mmc22.pdf]

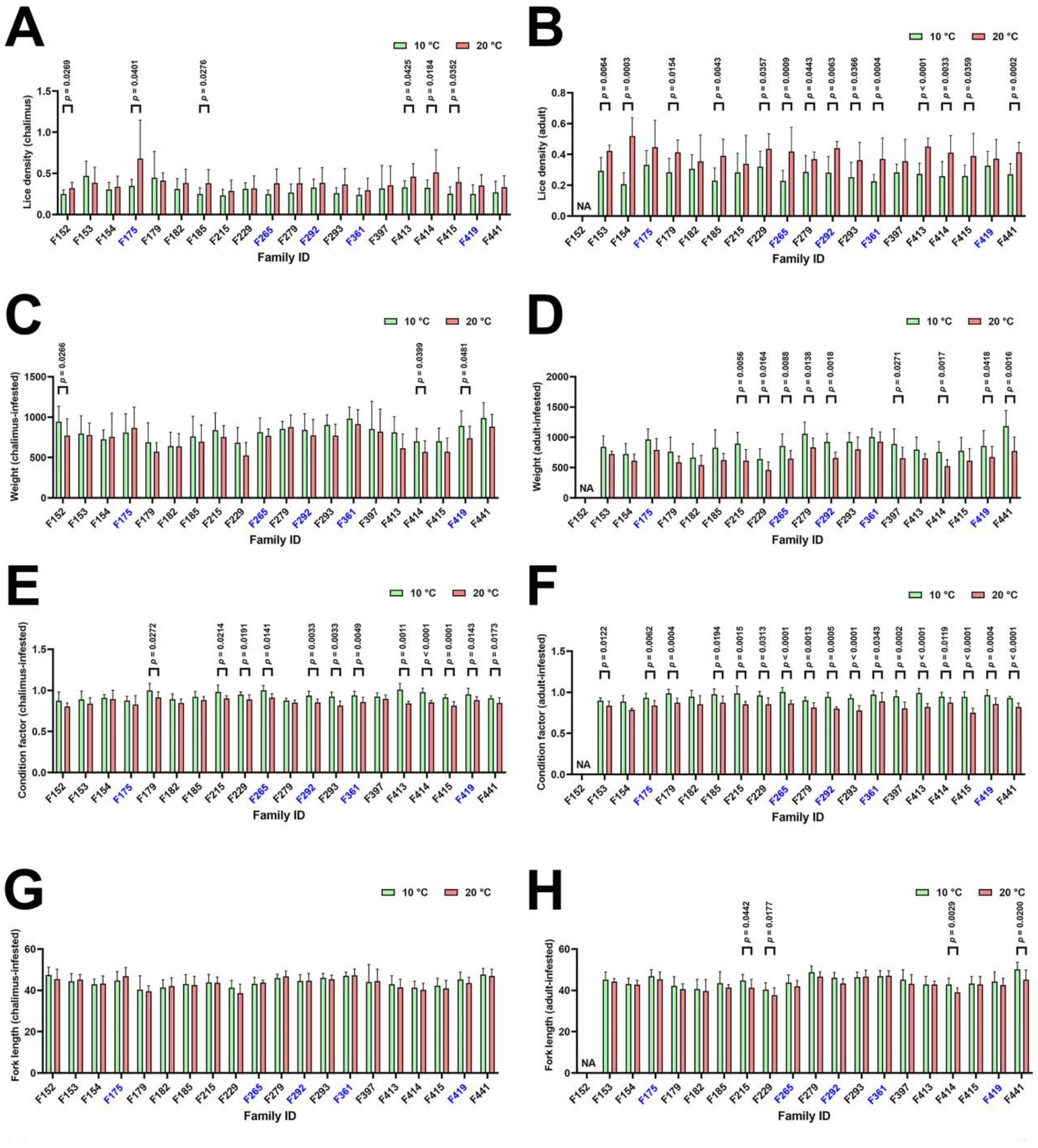

Supplementary Fig. 1. Comparison of mean lice density (MLD), mean body weight (MBW), mean condition factor (MCF) and mean fork length (MFL) between temperature conditions (20 °C vs. 10 °C) in salmon families infested with chalimus or adult stages of lice. (A) MLD in chalimus-infested families. (B) MLD in adult-infested families. (C) MBW in chalimus-infested families. (D) MBW in adult-infested families. (E) MCF in chalimus-infested families. (F) MCF in adult-infested families. (G) MFL in chalimus-infested families. (H) MFL in adult-infested families. Significant differences ($p < 0.05$) are indicated based on within-family comparisons

between temperature conditions. Families included in the RNA-seq analysis are indicated in blue font. Data are presented as mean ± SD.

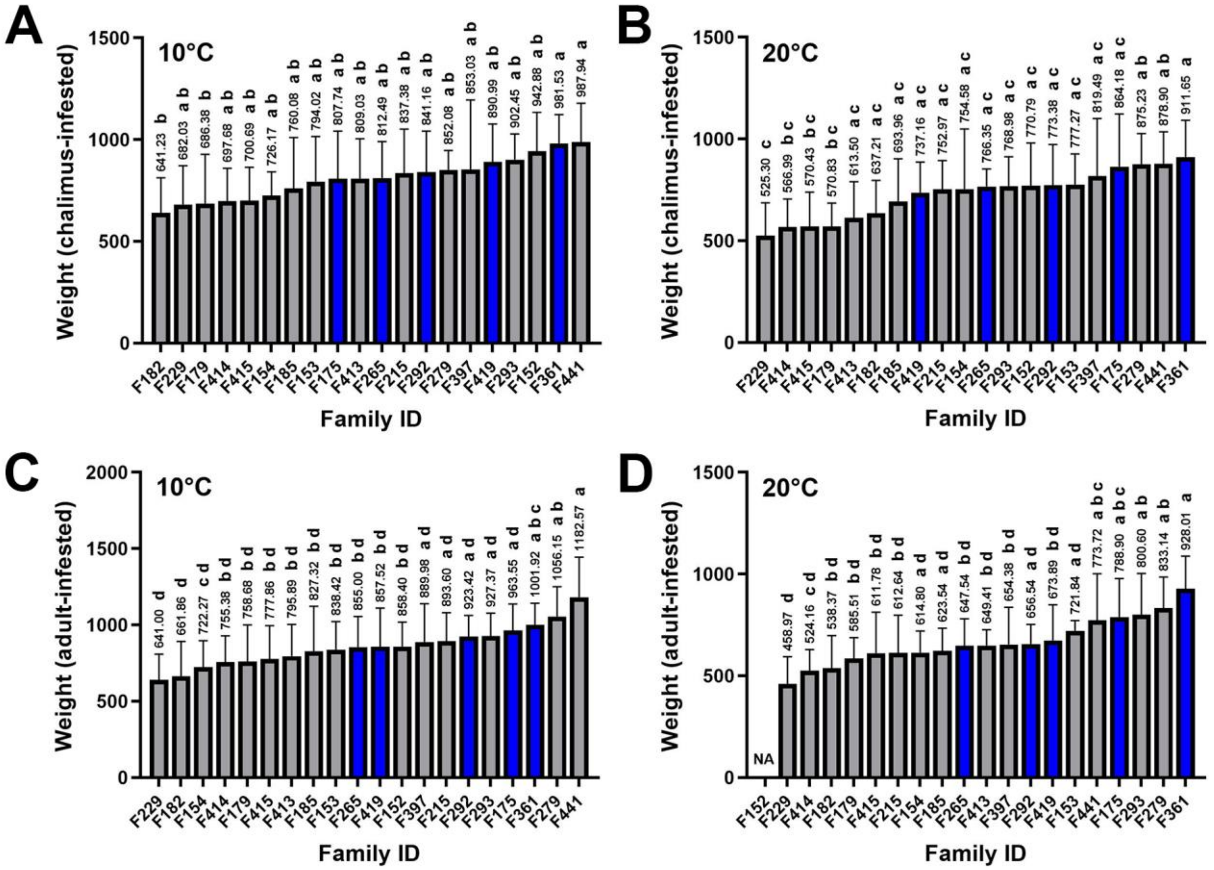

Supplementary Fig. 2. Summary of mean body weight (MBW, mean ± SD) in salmon families parasitized with chalimus or adult stages of lice under physiological (10 °C) and elevated (20 °C) temperature conditions. (**A**) MBW in chalimus-infested families at 10 °C. (**B**) MBW in chalimus-infested families at 20 °C. (**C**) MBW in adult-infested families at 10 °C. (**D**) MBW in adult-infested families at 20 °C. Different lowercase letters denote significant differences ($p < 0.05$) in MBW among families. MBW values are shown above the bars. Blue-highlighted bars indicate families selected for RNA-seq analysis.

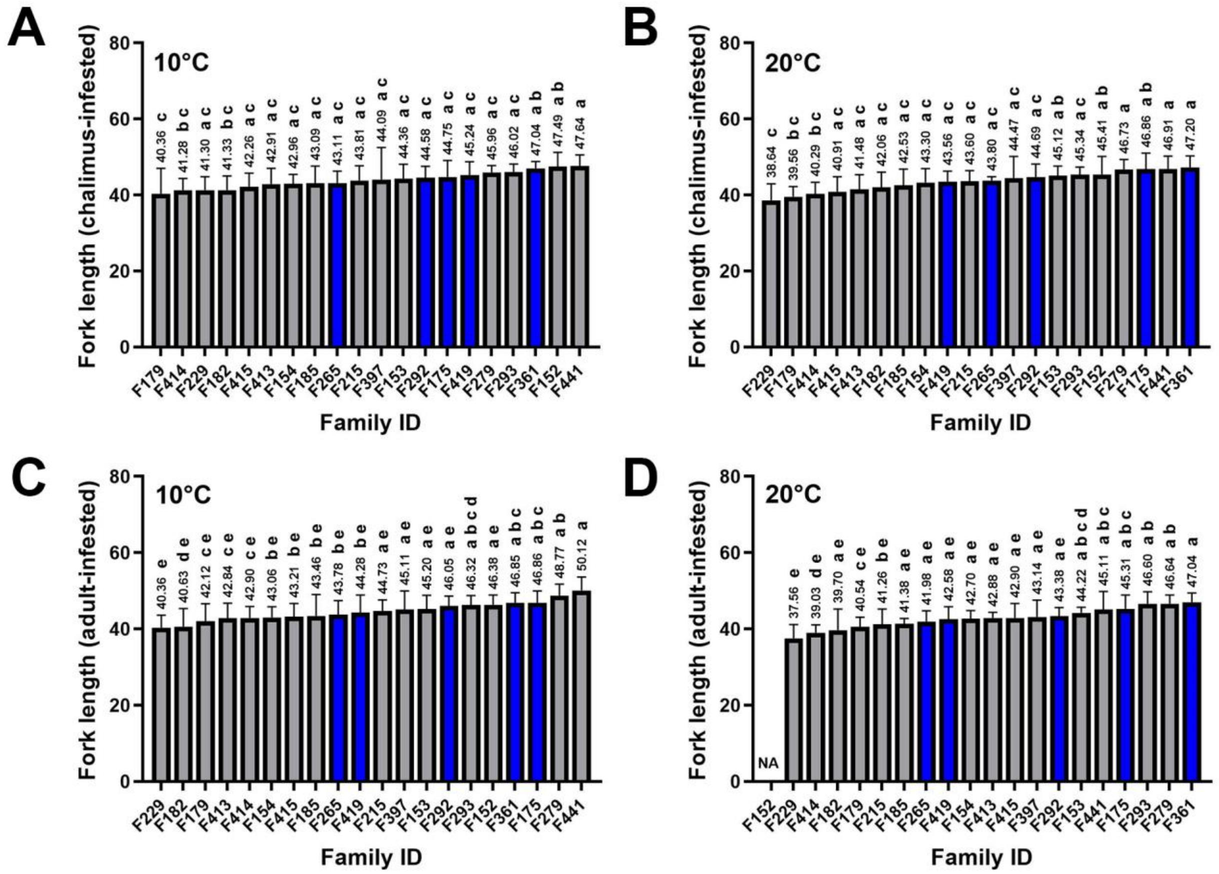

Supplementary Fig. 3. Summary of mean fork length (MFL, mean ± SD) in salmon families parasitized with chalimus or adult stages of lice under physiological (10 °C) and elevated (20 °C) temperature conditions. (**A**) MFL in chalimus-infested families at 10 °C. (**B**) MFL in chalimus-infested families at 20 °C. (**C**) MFL in adult-infested families at 10 °C. (**D**) MFL in adult-infested families at 20 °C. Different lowercase letters denote significant differences ($p < 0.05$) in MFL among families. MFL values are shown above the bars. Blue-highlighted bars indicate families selected for RNA-seq analysis.

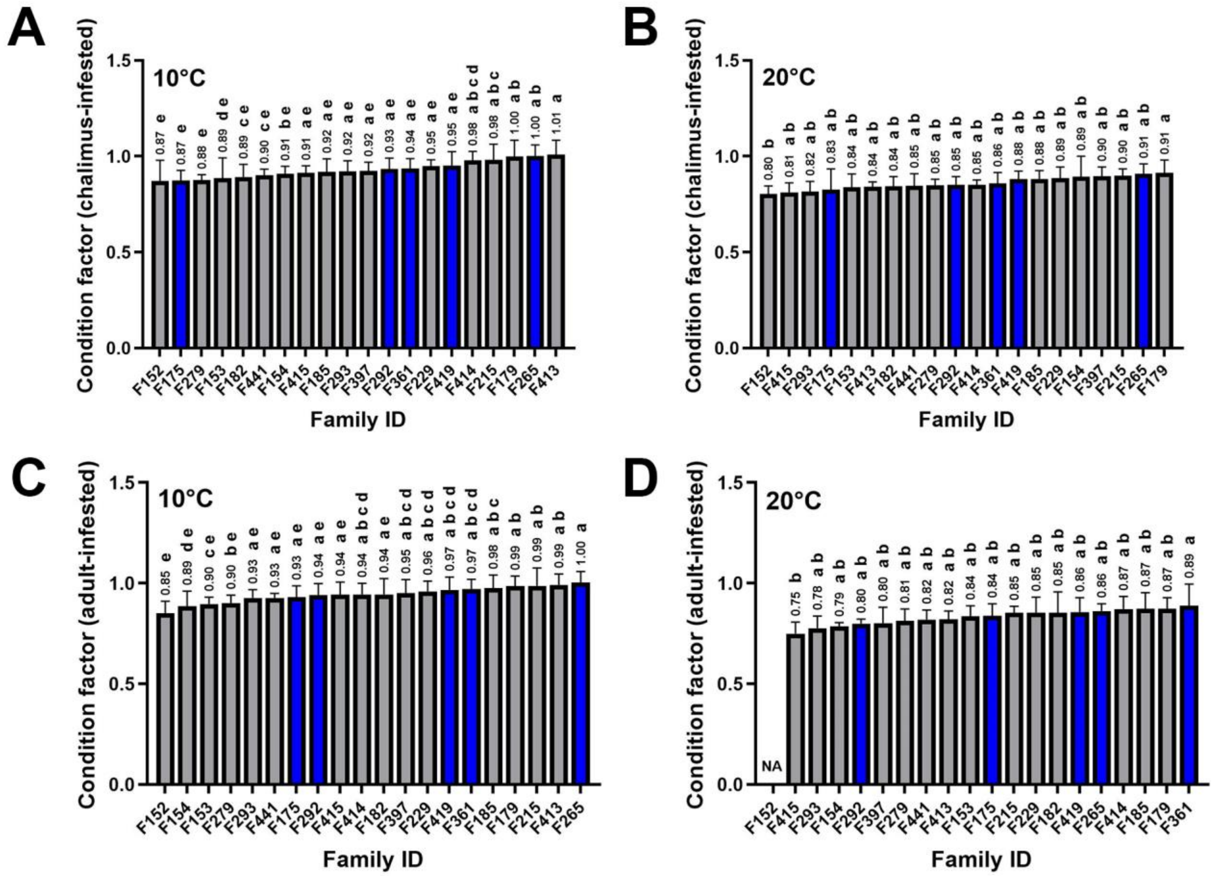

Supplementary Fig. 4. Summary of mean condition factor (MCF, mean ± SD) in salmon families parasitized with chalimus or adult stages of lice under physiological (10 °C) and elevated (20 °C) temperature conditions. (**A**) MCF in chalimus-infested families at 10 °C. (**B**) MCF in chalimus-infested families at 20 °C. (**C**) MCF in adult-infested families at 10 °C. (**D**) MCF in adult-infested families at 20 °C. Different lowercase letters denote significant differences ($p < 0.05$) in MCF among families. MCF values are shown above the bars. Blue-highlighted bars indicate families selected for RNA-seq analysis.

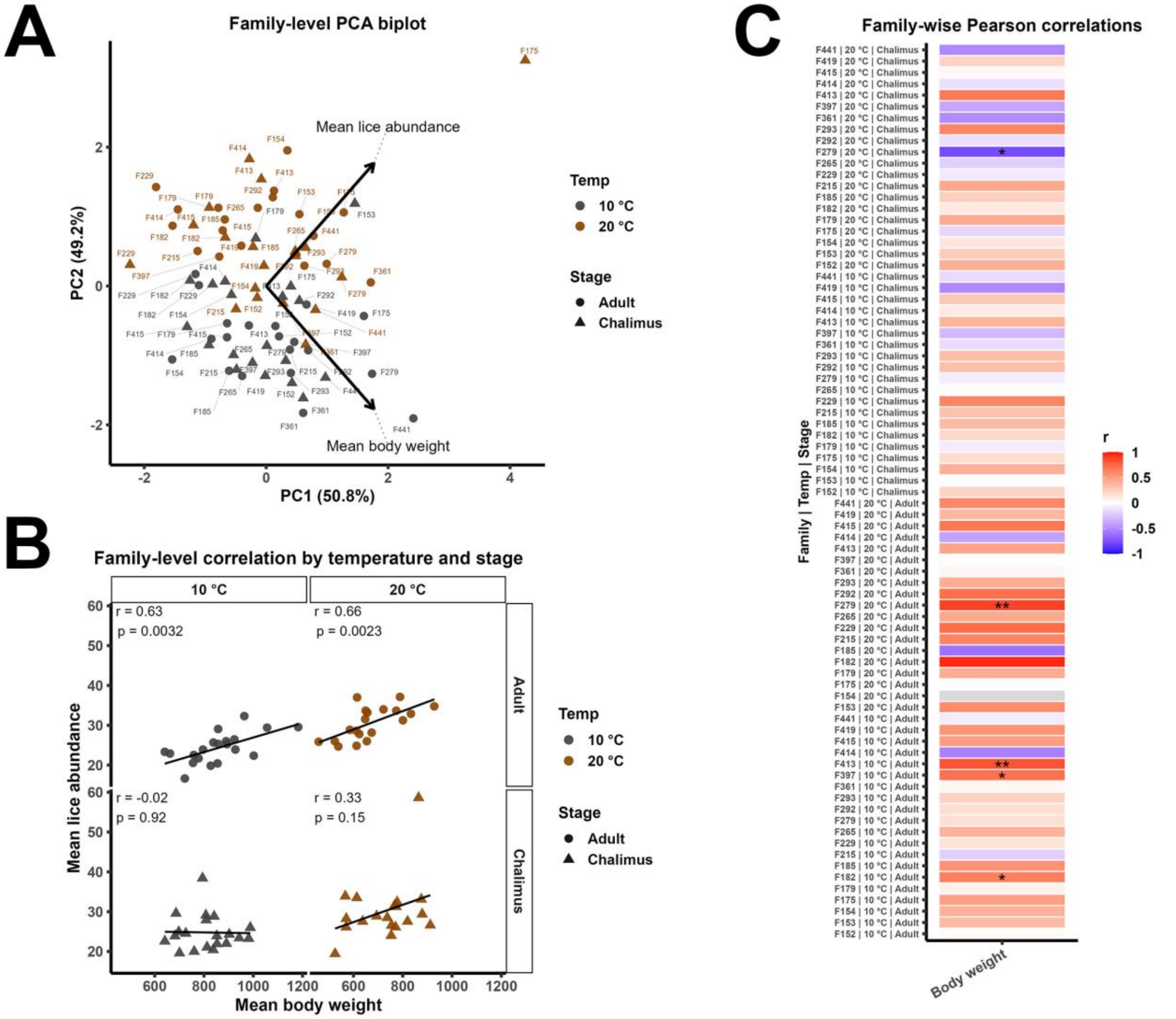

Supplementary Fig. 5. Principal component and correlation analyses of lice abundance and body weight in Atlantic salmon. (**A**) PCA biplot of family-level mean values showing relationships between lice abundance and body weight. Each point represents a family under a specific temperature and parasite developmental stage. Arrows indicate the direction and relative contribution of variables to the principal components. (**B**) Family-level Pearson correlation analyses between mean lice abundance and mean body weight. Analyses were performed separately for each temperature (10 °C and 20 °C) and parasite developmental stage (adult and chalimus). Colors represent temperature (dark grey: 10 °C; dark orange: 20 °C), and symbols represent parasite stage (circles: adult; triangles: chalimus). Pearson's correlation coefficients (*r*) and associated *p* values are shown within each panel. (**C**) Family-wise Pearson correlations between lice abundance and body weight in Atlantic salmon are shown as a heatmap of correlation coefficients (*r*) across temperatures (10 °C and 20 °C) and parasite stages (chalimus and adult). Color gradients represent the strength and direction of correlations (blue = negative, white = no correlation, red = positive). Asterisks indicate statistically significant correlations (* $p < 0.05$, ** $p < 0.01$). Rows represent individual family-temperature-stage combinations.

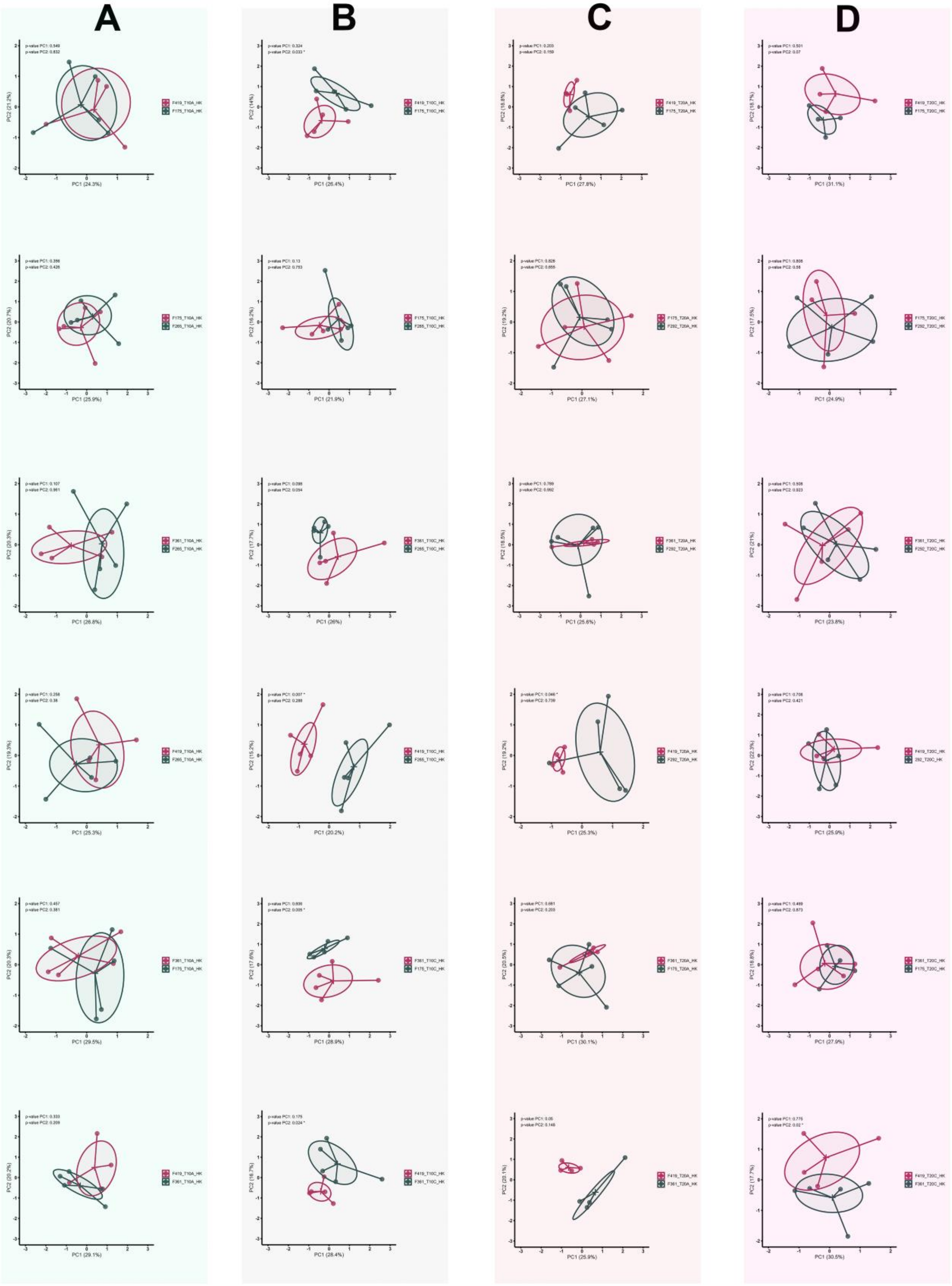
A
B
C
D

Supplementary Fig. 6. Principal Component Analysis (PCA) plots showing the distribution of RNA-seq samples based on raw count data from all expressed genes. The plots illustrate the distribution of head kidney samples from different salmon families infested with sea lice at varying stages and temperatures: (**A**) Adult stage lice at 10 °C. (**B**) Chalimus stage lice at 10 °C. (**C**) Adult stage lice at 20 °C. (**D**) Chalimus stage lice at 20 °C.

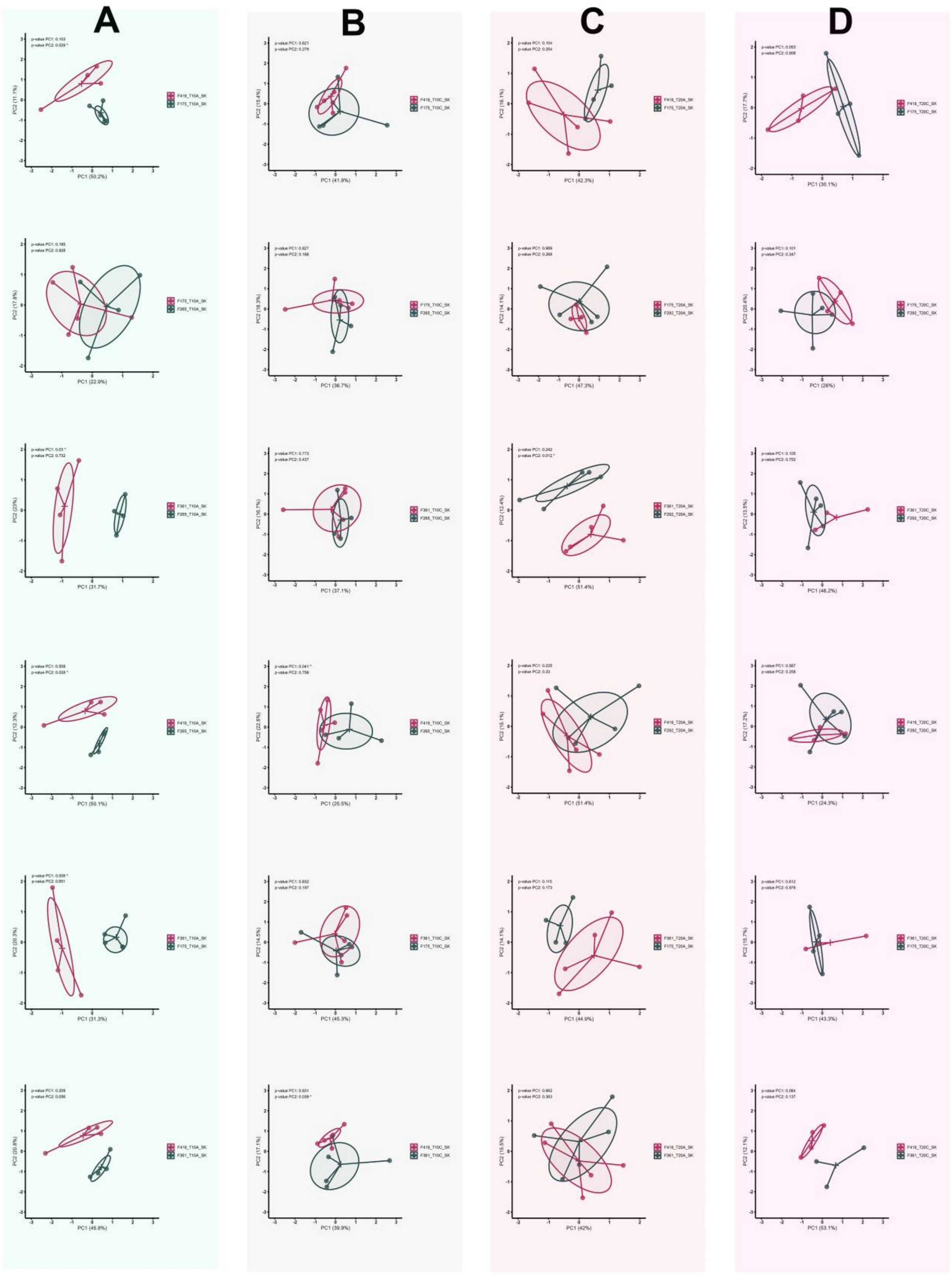
A
B
C
D

Supplementary Fig. 7. Principal Component Analysis (PCA) plots showing the distribution of RNA-seq samples based on raw count data from all expressed genes. The plots illustrate the distribution of skin samples from different salmon families infested with sea lice at varying stages and temperatures: (**A**) Adult stage lice at 10 °C. (**B**) Chalimus stage lice at 10 °C. (**C**) Adult stage lice at 20 °C. (**D**) Chalimus stage lice at 20 °C.

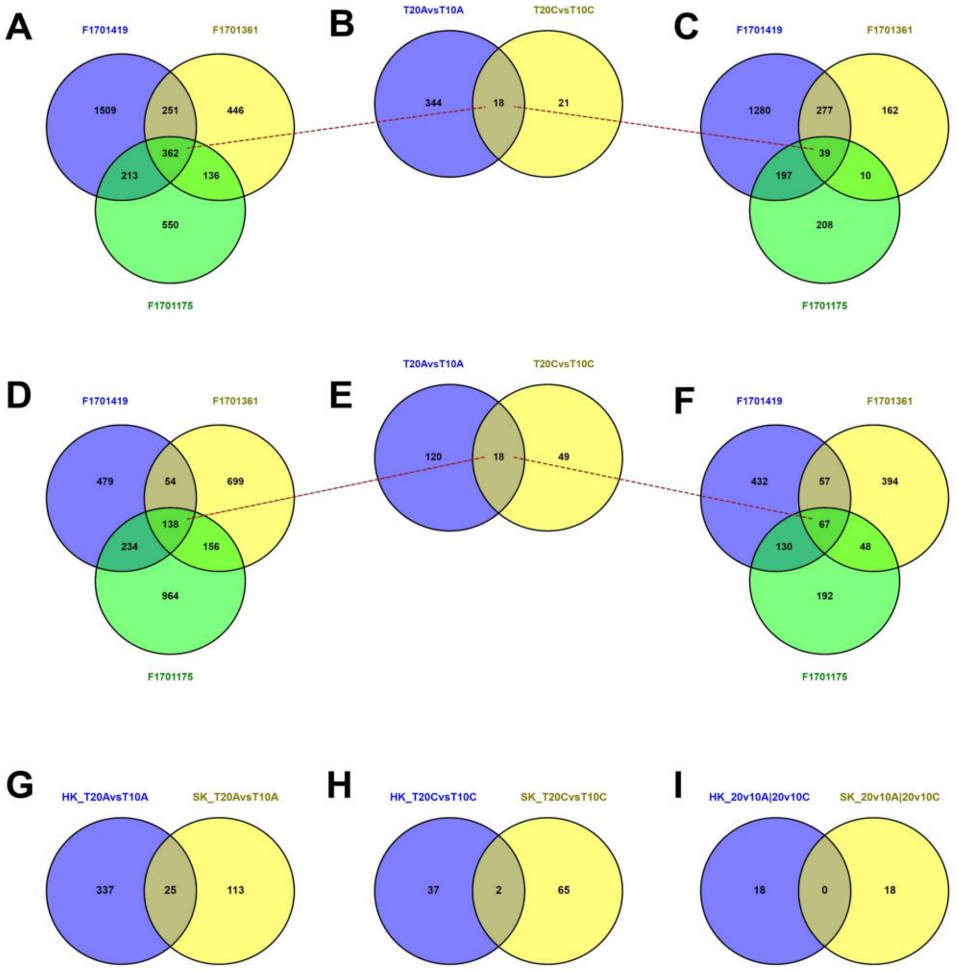

Supplementary Fig. 8. Head kidney and skin transcriptome responses of Atlantic salmon infested with adult or chalimus stages of sea lice at elevated (20 °C) compared to normal (10 °C) temperature conditions. (**A-C**) Venn diagrams showing the distribution of DEGs identified based on comparisons of head kidney samples of specific families infested with adult (**A**) or chalimus (**C**) stages of lice at 20 °C versus 10 °C, along with DEGs shared between families infested by adult and those parasitized with chalimus stages of lice (**B**). (**D-F**) Venn diagrams showing the distribution of DEGs identified based on comparisons of skin samples of specific families infested with adult (**D**) or chalimus (**F**) stages of lice at 20 °C versus 10 °C, along with DEGs shared between families infested by adult and those parasitized with chalimus stages of lice (**E**). (**G-I**) Venn diagrams showing the distribution of DEGs shared between head kidney and skin tissues of specific salmon families infested with adult (**G**) or chalimus (**H**) stages of lice at 20 °C versus 10 °C, with no shared DEGs between both tissues when DEGs shared between families infested by adult and those parasitized with chalimus stages of lice were examined (**I**).

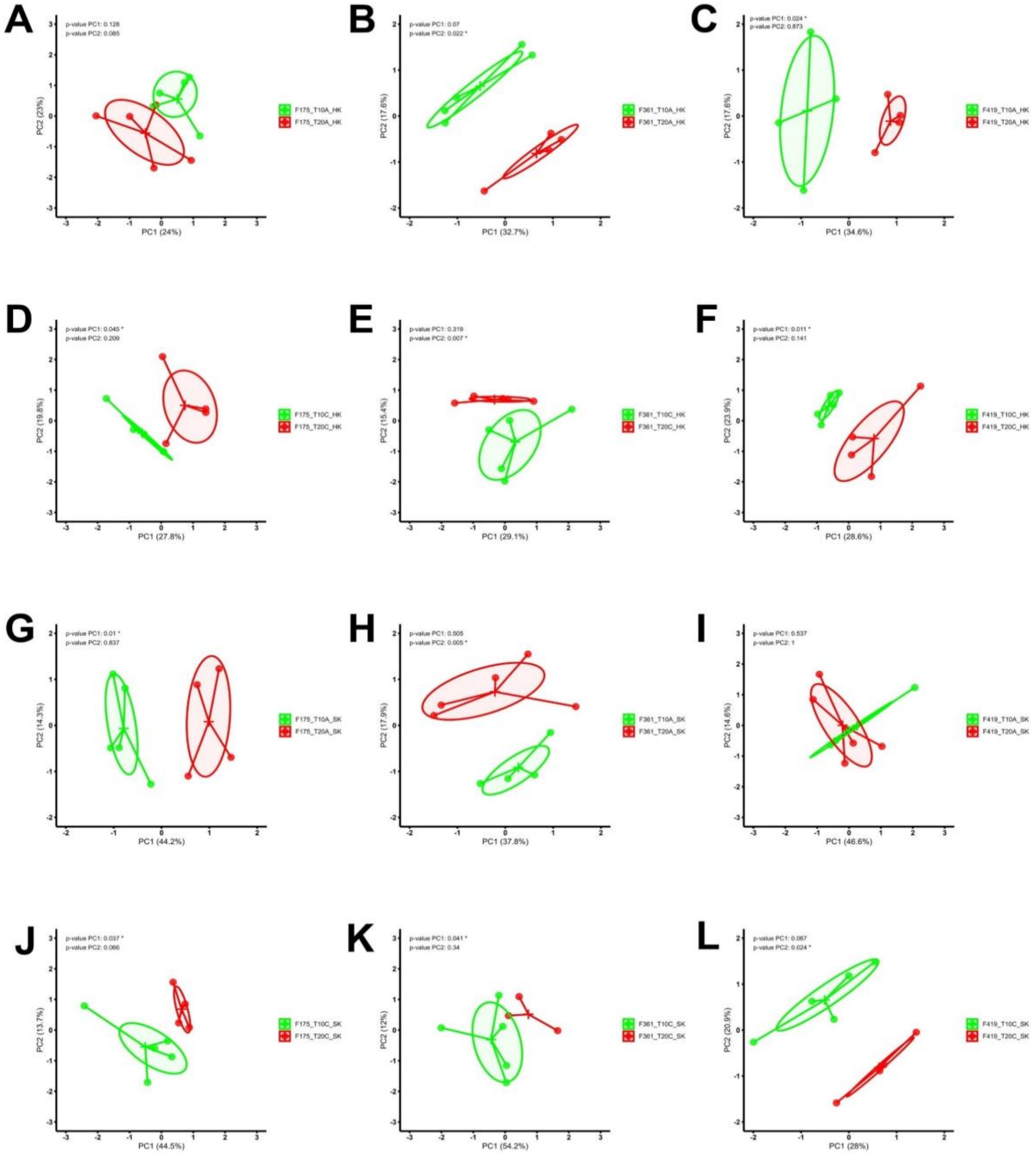

Supplementary Fig. 9. Principal Component Analysis (PCA) plots illustrating the distribution of RNA-seq samples based on raw count data from all expressed genes. These plots compare samples between 20 °C and 10 °C conditions: (**A-C**) head kidney samples from specific families infested with adult lice, (**D-F**) head kidney samples from specific families infested with chalimus lice, (**G-I**) skin samples from specific families infested with adult lice, and (**J-L**) skin samples from specific families infested with chalimus lice.

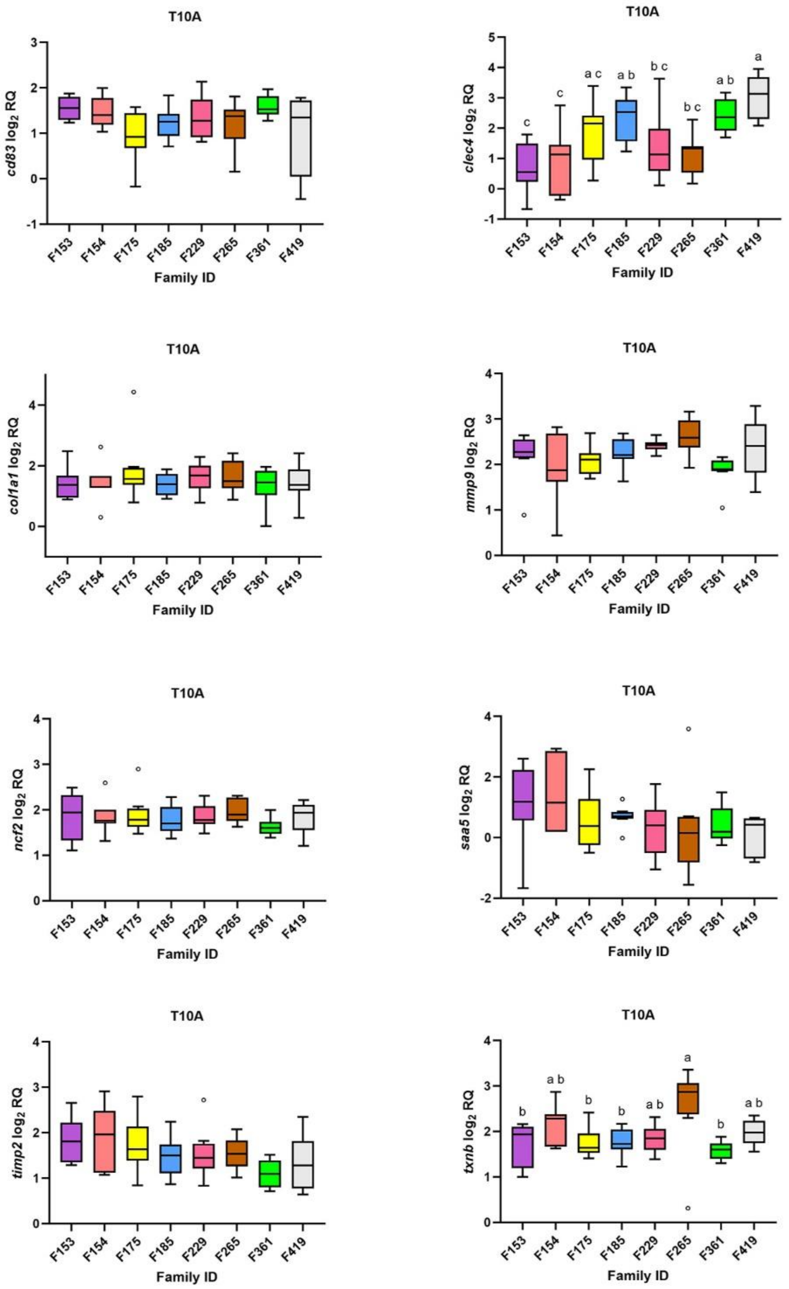

Supplementary Fig. 10. qPCR results for tested GOIs (*cd83*, *clec4*, *col1a1*, *mmp9*, *ncf2*, *saa5*, *timp2* and *txnb*) in the head kidney of different families infested by adult stages of sea lice at 10 °C. Data are presented as box plots with median and Tukey whiskers. Different lowercase letters denote significant differences ($p < 0.05$) in gene expression responses among families.

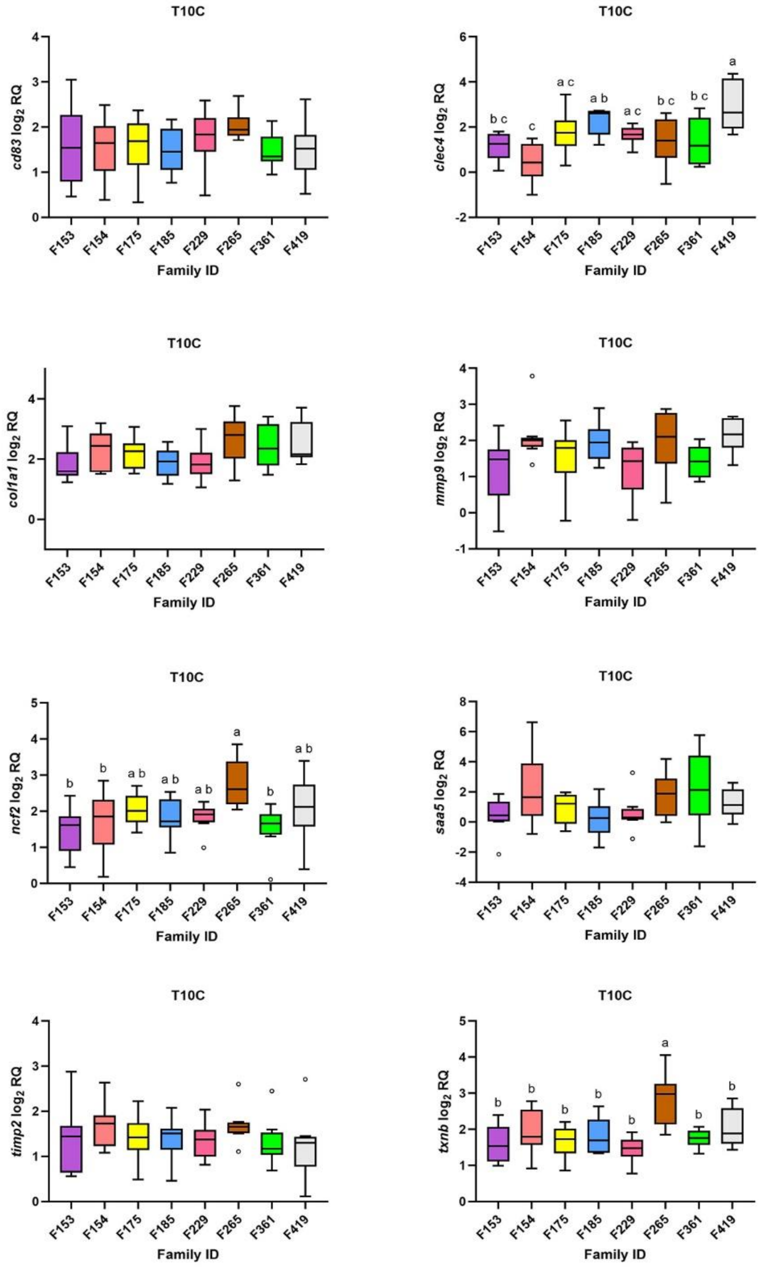

Supplementary Fig. 11. qPCR results for tested GOIs (*cd83*, *clec4*, *col1a1*, *mmp9*, *ncf2*, *saa5*, *timp2* and *txnb*) in the head kidney of different families infested by chalimus stages of sea lice at 10 °C. Data are presented as box plots with median and Tukey whiskers. Different lowercase letters denote significant differences ($p < 0.05$) in gene expression responses among families.

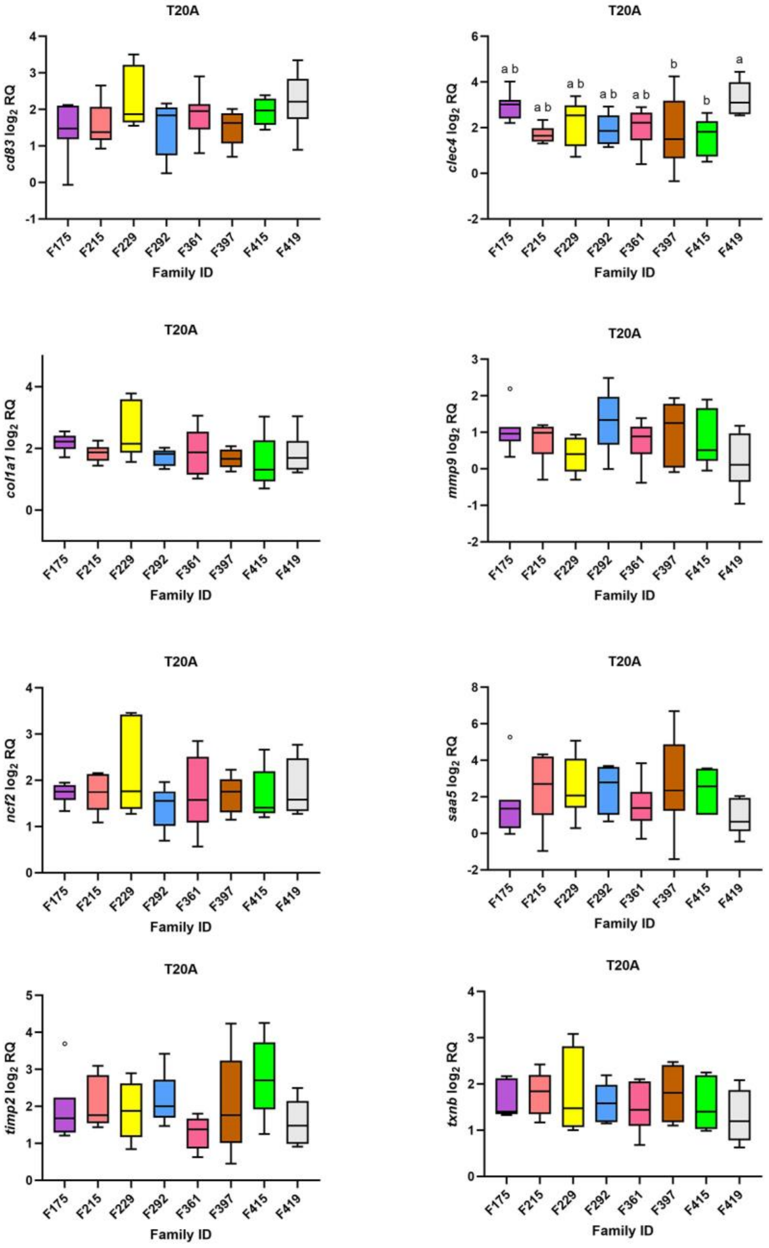

Supplementary Fig. 12. qPCR results for tested GOIs (*cd83*, *clec4*, *col1a1*, *mmp9*, *ncf2*, *saa5*, *timp2* and *txnb*) in the head kidney of different families infested by adult stages of sea lice at 20 °C. Data are presented as box plots with median and Tukey whiskers. Different lowercase letters denote significant differences ($p < 0.05$) in gene expression responses among families.

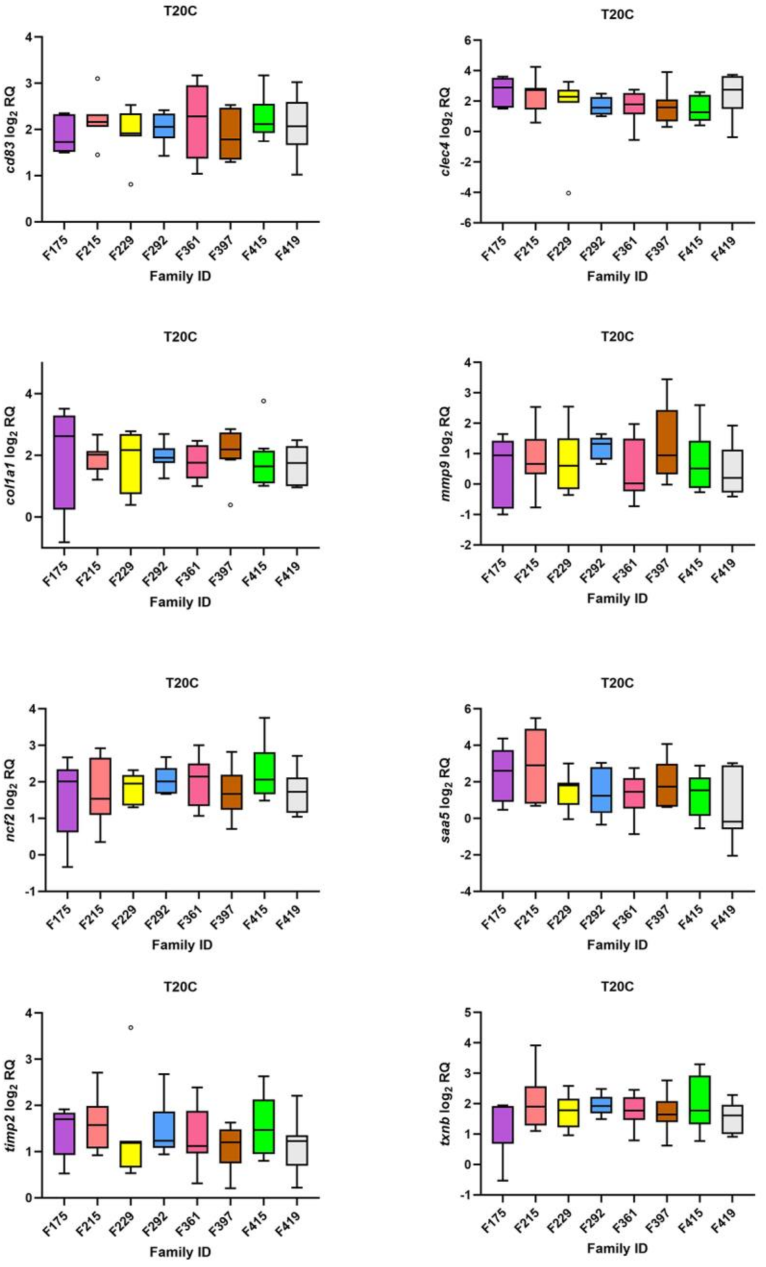

Supplementary Fig. 13. qPCR results for tested GOIs (*cd83*, *clec4*, *col1a1*, *mmp9*, *ncf2*, *saa5*, *timp2* and *txnb*) in the head kidney of different families infested by chalimus stages of sea lice at 20 °C. Data are presented as box plots with median and Tukey whiskers. Different lowercase letters denote significant differences ($p < 0.05$) in gene expression responses among families.

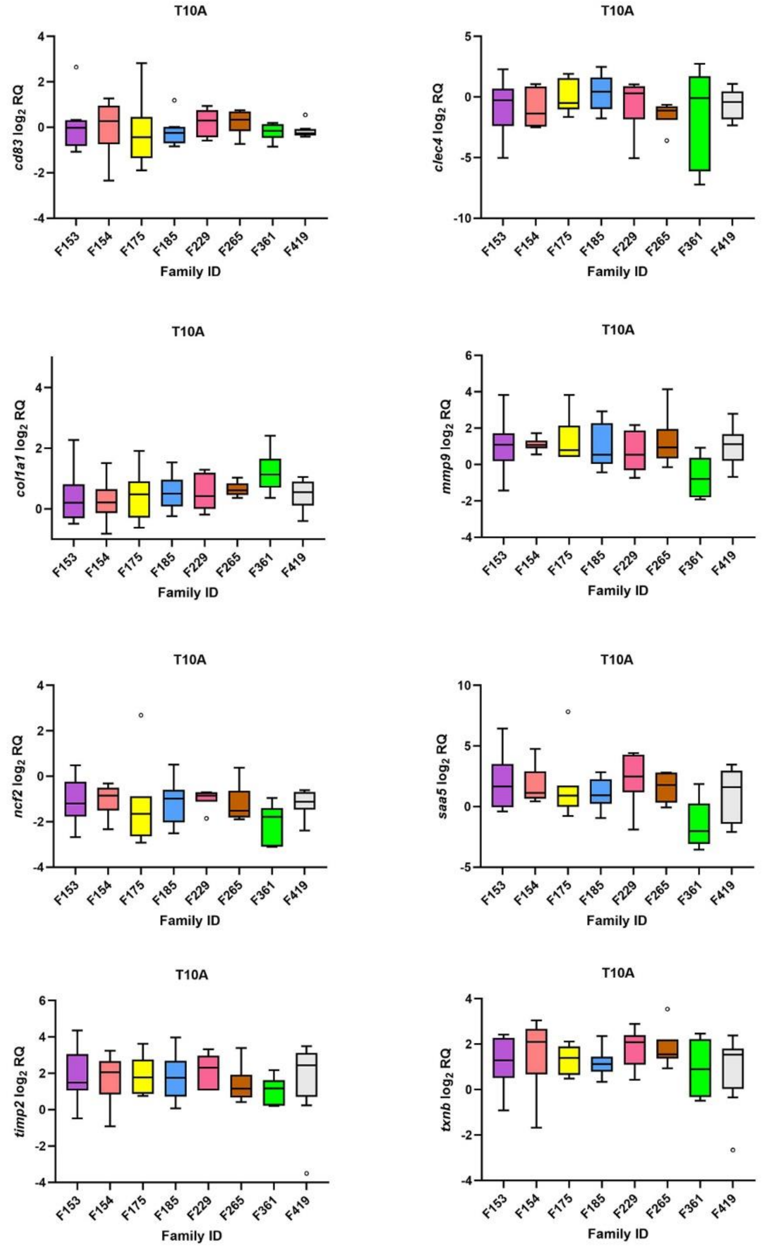

Supplementary Fig. 14. qPCR results for tested GOIs (*cd83*, *clec4*, *col1a1*, *mmp9*, *ncf2*, *saa5*, *timp2* and *txnb*) in the skin of different families infested by adult stages of sea lice at 10 °C. Data are presented as box plots with median and Tukey whiskers. Different lowercase letters denote significant differences ($p < 0.05$) in gene expression responses among families.

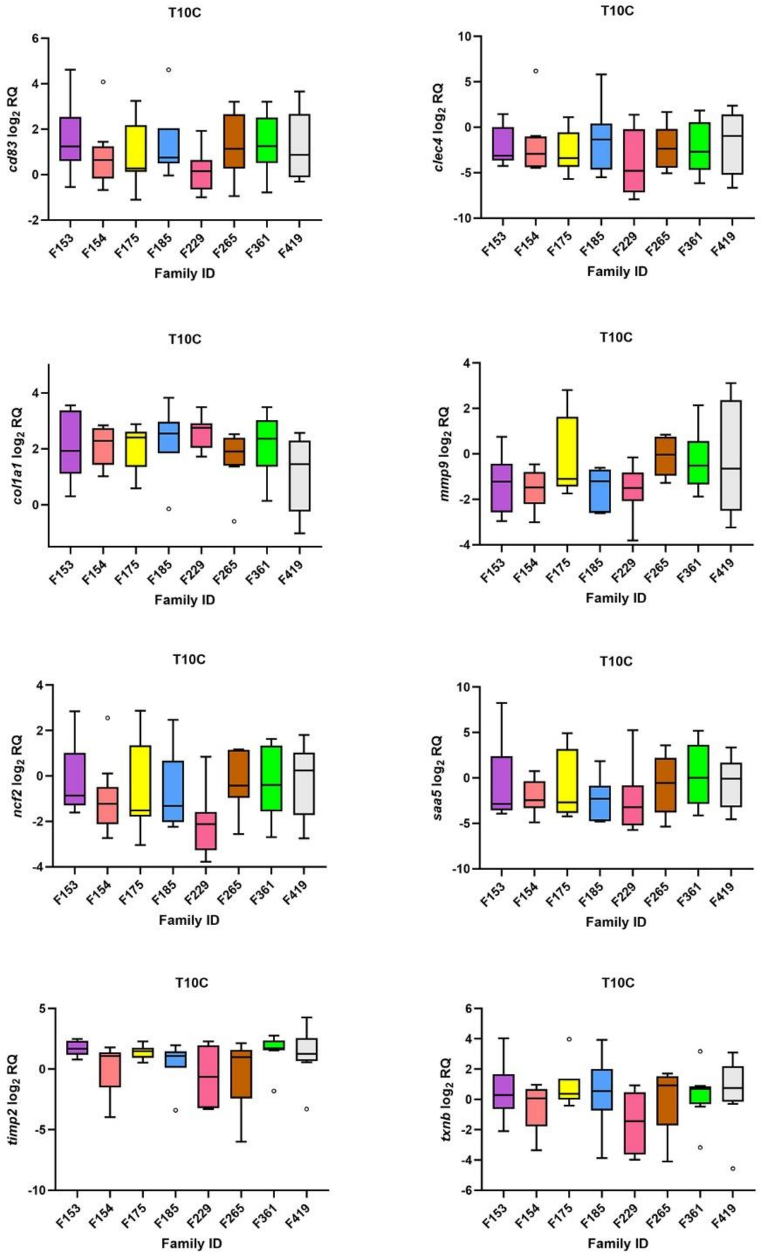

Supplementary Fig. 15. qPCR results for tested GOIs (*cd83*, *clec4*, *col1a1*, *mmp9*, *ncf2*, *saa5*, *timp2* and *txnb*) in the skin of different families infested by chalimus stages of sea lice at 10 °C. Data are presented as box plots with median and Tukey whiskers. Different lowercase letters denote significant differences ($p < 0.05$) in gene expression responses among families.

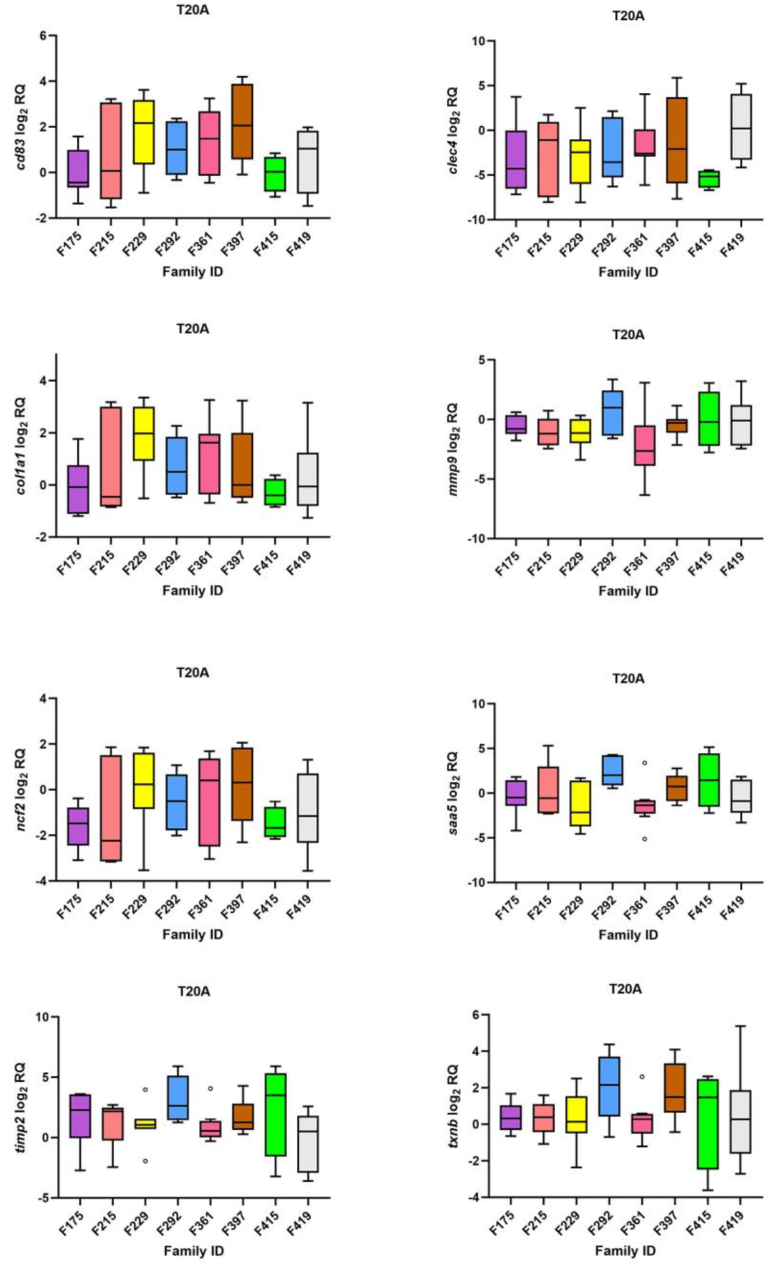

Supplementary Fig. 16. qPCR results for tested GOIs (*cd83*, *clec4*, *col1a1*, *mmp9*, *ncf2*, *saa5*, *timp2* and *txnb*) in the skin of different families infested by adult stages of sea lice at 20 °C. Data are presented as box plots with median and Tukey whiskers. Different lowercase letters denote significant differences ($p < 0.05$) in gene expression responses among families.

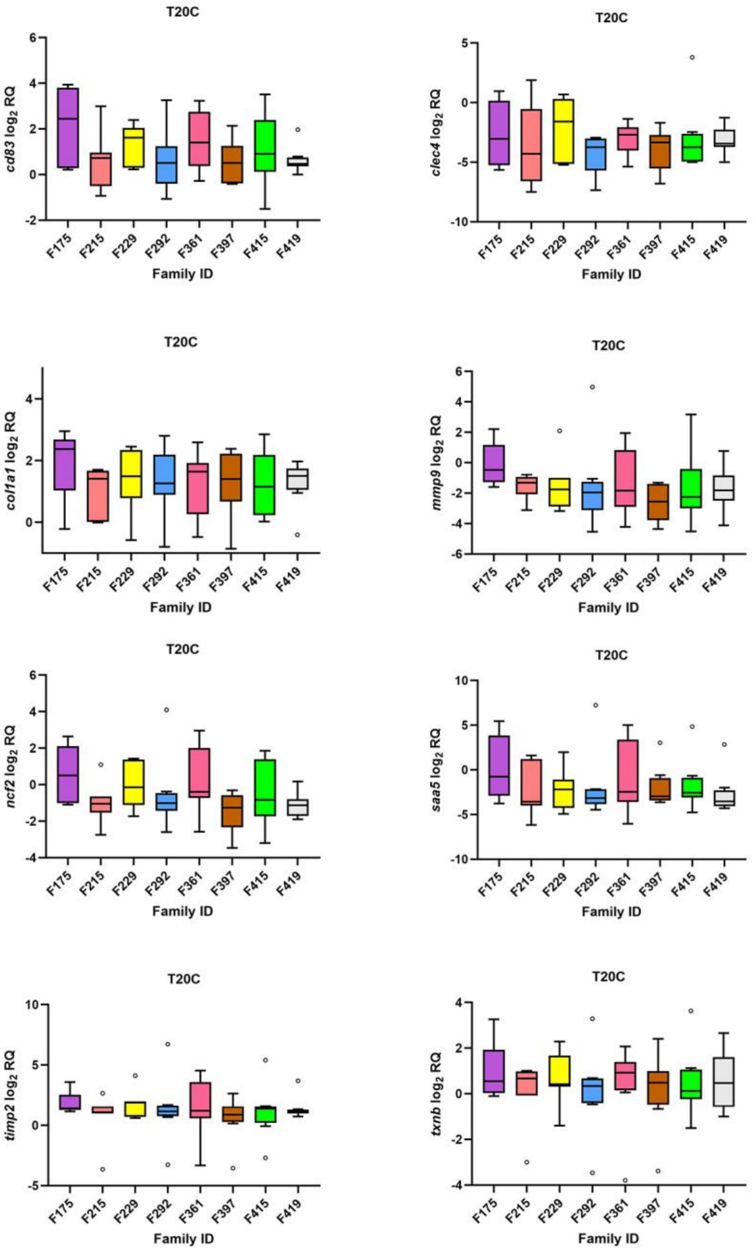

Supplementary Fig. 17. qPCR results for tested GOIs (*cd83*, *clec4*, *col1a1*, *mmp9*, *ncf2*, *saa5*, *timp2* and *txnb*) in the skin of different families infested by chalimus stages of sea lice at 20 °C. Data are presented as box plots with median and Tukey whiskers. Different lowercase letters denote significant differences ($p < 0.05$) in gene expression responses among families.

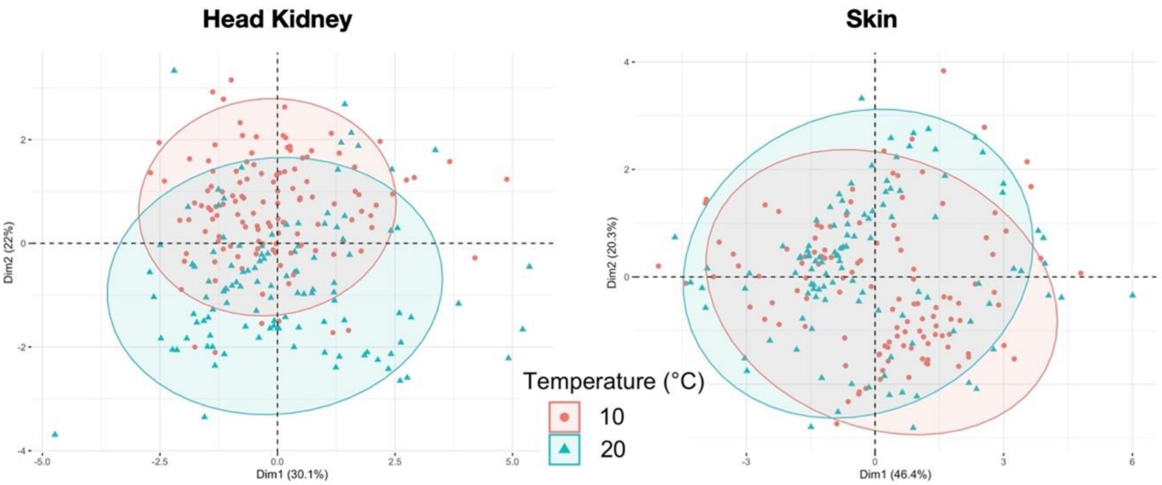

Supplementary Fig. 18. Principal components analysis (PCA) illustrating the expression data distribution of qPCR data for tested GOIs (*cd83*, *clec4*, *col1a1*, *mmp9*, *ncf2*, *saa5*, *timp2* and *txnb*) in head kidney (left) and skin (right) tissues, with ellipses covering 95% of a normal distribution for 10 °C (red) and 20 °C (blue) groups.

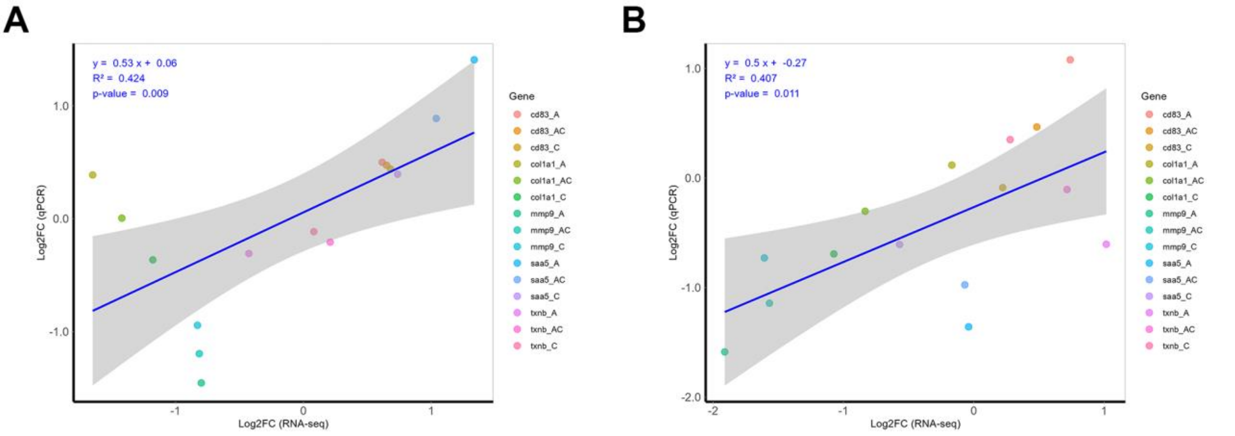

Supplementary Fig. 19. Relationship between log_2-transformed fold-changes (FC) of the qPCR and RNA-seq datasets. Scatterplot of log_2-transformed gene expression fold-changes (FC) calculated from the mean values of qPCR (normalized to reference genes) and RNA-seq datasets (TMM-normalized) for selected genes with significant changes based on temperature differences (20 °C compared to 10 °C) in the head kidney (**A**) and skin (**B**). Figure keys: adult (A), chalimus (C) and adult + chalimus (AC).

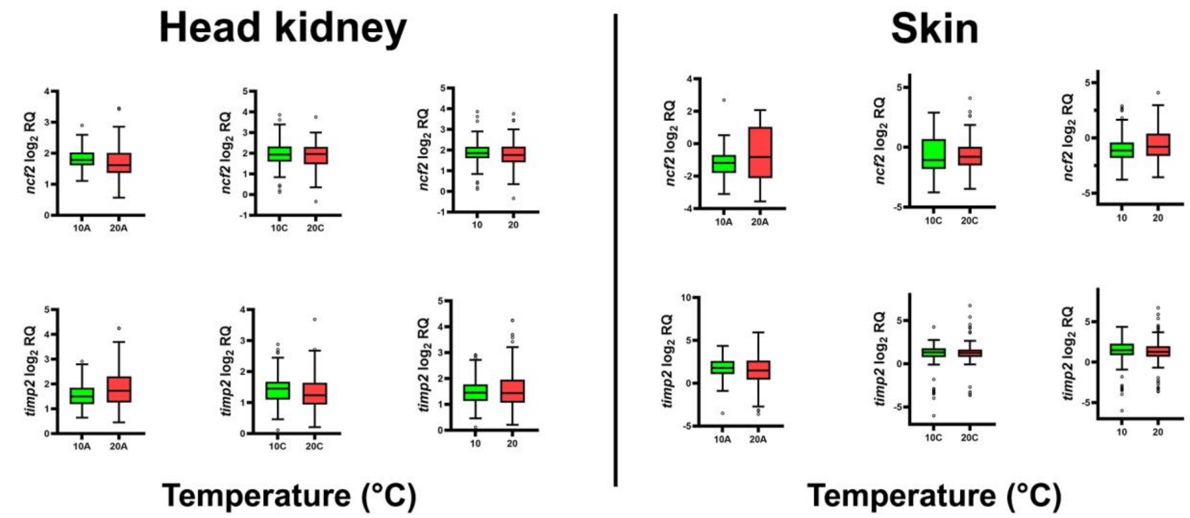

Supplementary Fig. 20. qPCR results for *ncf2* and *timp2* in the head kidney and skin of salmon families infested under elevated (20 °C) and normal (10 °C) temperature conditions. Data are presented as box plots with median values and Tukey whiskers. Significant differences ($p < 0.05$) in gene expression responses between families under the two temperature conditions are indicated by asterisks. For each gene, expression responses to temperature were analyzed in salmon infested with adult lice (left plot), chalimus lice (middle plot), and across all infested salmon regardless of stage (right plot).

Supplementary Table 1. Selected families (n=5) included in RNA sequencing analyses.

Family ID	Temperature		Infestation stage	
	10 °C	20 °C	chalimus	adult
F419	+	+	+	+
F361	+	+	+	+
F175	+	+	+	+
F265	+	–	+	+
F292	–	+	+	+

Supplementary Table 2. The mean lice density (MLD ± SD) and mean lice abundance (MLA ± SD) in salmon families infested by chalimus or adult stages of lice at 10 °C and 20 °C (included in RNA sequencing analyses).

Family ID	10 °C				20 °C			
	Chalimus-infested		Adult-infested		Chalimus-infested		Adult-infested	
	MLD	MLA	MLD	MLA	MLD	MLA	MLD	MLA
F419	0.250 ± 0.110	22.00 ± 7.29	0.326 ± 0.094	29.10 ± 8.77	0.352 ± 0.132	28.50 ± 11.07	0.371 ± 0.126	28.15 ± 10.08
F361	0.239 ± 0.078	23.25 ± 6.82	0.225 ± 0.045	22.36 ± 3.93	0.295 ± 0.146	26.62 ± 10.67	0.370 ± 0.135	34.78 ± 11.52
F175	0.347 ± 0.080	29.10 ± 6.03	0.331 ± 0.094	32.30 ± 10.24	0.680 ± 0.466	58.60 ± 35.11	0.447 ± 0.175	37.12 ± 12.64
F265	0.248 ± 0.051	21.10 ± 3.39	0.228 ± 0.068	20.42 ± 6.93	0.380 ± 0.173	31.50 ± 13.33	0.419 ± 0.157	31.54 ± 13.14
F292	0.327 ± 0.103	28.82 ± 9.99	0.281 ± 0.105	26.45 ± 9.95	0.384 ± 0.185	31.25 ± 13.82	0.440 ± 0.044	33.20 ± 4.66

Supplementary Table 3. Differentially expressed genes (DEGs) obtained based on pairwise comparisons within specific salmon families parasitized with chalimus or adult stages of lice at two different temperature conditions for each tissue (for families with RNA-seq data at both temperatures).

Pairwise comparisons	No. of DEGs in skin			No. of DEGs in head kidney		
	Up	Down	Total	Up	Down	Total
T20A vs. T10A						
F419	620	285	905	1410	925	2335
F361	434	613	1047	587	608	1195
F175	911	581	1492	854	407	1261
Shared DEGs			138			362
T20C vs. T10C						
F419	195	491	686	801	992	1793
F361	341	225	566	133	355	488
F175	165	272	437	326	128	454
Shared DEGs			67			39
T20A vs. T10A | T20C vs. T10C						
Shared DEGs			18			18

Supplementary Table 4. Summary of leading GO terms/KEGG pathways enriched based on shared DEGs related to pairwise comparisons between all families parasitized with adult or chalimus stages of lice at 20 °C versus 10 °C (T20A vs. T10A | T20C vs. T10C) in the head kidney.

**GO Description (L)**	**GO/KEGG ID (L + SL)**	**Gene ID**	**Gene symbol**	**Gene description**	**Fold-change (log_2FC)**	
					T20A vs. T10A (all families)	T20C vs. T10C (all families)
Cysteine-type endopeptidase inhibitor activity	GO:0004869	LOC100195750	*salarin*	Salarin	3.385395	1.942499
		LOC106590538	*lcn*	Lipocalin	1.964349	1.379509
		LOC106589915	*cst*	Cystatin	1.35151	1.291867
Defense response to bacterium	GO:0042742	*hamp*	*hamp*	Hepcidin antimicrobial peptide	2.778781	2.203403
		lyz	*lyz*	Lysozyme	1.861199	1.418188
		tfa	*tfa*	Transferrin-a	1.602798	1.654987
		LOC106564853	*epx*	Eosinophil peroxidase	-3.10406	-1.38847
Neuron apoptotic process	GO:0051402	*apoeb*	*apoeb*	Apolipoprotein Eb	3.658369	2.538333
		m17	*il6*	IL-6 subfamily cytokine M17	2.174343	1.79393
		hdr	—	Hematopoietic death receptor	-1.3366	-1.5078
Ammonium transmembrane transporter activity	GO:0072488; GO:0008519	LOC106600435	*aqpa*	Aquaporin FA-CHIP	-1.84931	-1.40969
		LOC100196095	*rhbg*	Rhesus blood group, B glycoprotein	-2.03075	-1.55831
		rhd	*rhd*	Rh blood group, D antigen	-2.89066	-1.68335
Glyoxylate and dicarboxylate metabolism	KEGG:00380; KEGG:00630	*glyctk*	*glyctk*	Glycerate kinase	3.421706	1.619655
		LOC106577031	*ido2*	Indoleamine 2,3-dioxygenase 2	2.025267	1.813782
		LOC106564824	*cat*	Catalase	-1.26298	-1.30092
		LOC106586175	*cat*	Catalase	-1.61779	-1.57397
Oxidoreductase activity, acting on single donors with incorporation of molecular oxygen, incorporation of two atoms of oxygen	GO:0016701; GO:0016702	*cdo1*	*ido2*	Cysteine dioxygenase type 1	4.322272	3.402714
		LOC106590602	*hgd*	Homogentisate 1,2-dioxygenase	2.11657	1.271435
		LOC106577031	*ido2*	Indoleamine 2,3-dioxygenase 2	2.025267	1.813782
Hemoglobin binding	GO:0030492; GO:0031721	LOC106601074	*hbb1*	Hemoglobin subunit beta-1	-2.90168	-1.78408
		LOC106607236	*hbb1*	Hemoglobin subunit beta-1	-3.09796	-3.2593

		LOC106607371	*hbb1*	Hemoglobin subunit beta-1	-3.15182	-1.53555
		LOC106607380	*hbb1*	Hemoglobin subunit beta-1	-3.8194	-1.61304
Chemokine activity	GO:0042379; GO:0008009	LOC106600447	*ccl4*	C-C motif chemokine 4	3.77808	1.997472
		cxcl10	*cxcl10*	C-X-C motif chemokine 10	2.783441	1.820649
		LOC106561115	*ccl4*	C-C motif chemokine 4	1.391794	1.518662
Myofibril assembly	GO:0055002; GO:0030239	LOC106569441	—	Putative leucine-rich repeat-containing protein DDB_G0290503	2.651918	1.62549
		LOC106589620	*klf1*	Kruppel-like factor 1	-2.44279	-1.9683
		tmod4	*tmod4*	Tropomodulin 4 (muscle)	-2.93724	-2.02441
		LOC106580917	*tmod4*	Tropomodulin-4	-3.32738	-2.15244
Tumor necrosis factor-mediated signaling pathway	GO:0034612; GO:0033209; GO:0071356	*hdr*	—	Hematopoietic death receptor	-1.3366	-1.5078
		traf4a	*traf4a*	TNF receptor-associated factor 4a	-1.34268	-1.33147
		LOC106563020	*traf2*	TNF receptor-associated factor 2	-1.40057	-1.37457
Iron ion transport	GO:0006879; GO:0000041; GO:0006826; GO:0008198	*cdo1*	*ido2*	Cysteine dioxygenase type 1	4.322272	3.402714
		hamp	*hamp*	Hepcidin antimicrobial peptide	2.778781	2.203403
		tfa	*tfa*	Transferrin-a	1.602798	1.654987
		LOC123741664	*ftm*	Ferritin, middle subunit	1.543516	1.25363
		LOC106600953	*ftm*	Ferritin, middle subunit	1.483139	1.299958
		slc39a4	*slc39a4*	Solute carrier family 39 member 4	-2.17046	-1.6271
		tfr1a	*tfr1a*	Transferrin receptor 1a	-2.5038	-1.71211
		slc25a37	*slc25a37*	Solute carrier family 25 member 37	-2.95293	-1.81116
ATP-dependent protein folding chaperone	GO:0009266; GO:0009408; GO:0044183; GO:0140662	*hs90a*	*hsp90aa1*	Heat shock protein HSP 90-alpha	3.905346	1.67078
		LOC106608136	*hsp90aa1*	Heat shock protein HSP 90-alpha	3.209955	1.884815
		hsp7c	*hspa8*	Heat shock cognate 71 kDa protein	2.951804	1.543754
		hsc70	*hspa8*	Heat shock cognate 70	2.606874	1.28164
		dnaja	*dnaja*	DnaJ heat shock protein family (Hsp40) member A	2.376474	1.375533
Lymphocyte mediated immunity	GO:0002443; GO:0009988; GO:0141061; GO:0001909; GO:0031640; GO:0002449; GO:0002460;	LOC106577762	*prf1*	Perforin-1	2.115216	1.403061
		lyz	*lyz*	Lysozyme	1.861199	1.418188
		LOC106577765	*prf1*	Perforin-1	1.770935	1.442441
		LOC106588291	*clec4e*	C-type lectin domain family 4 member E	1.569631	1.337551
		LOC106583003	*cd55*	Complement decay-accelerating factor	-1.70698	-1.34709
		spaca4l	*spaca4l*	Sperm acrosome associated 4 like	-1.87344	-1.37114

	GO:0002456					
Beta-Alanine metabolism	KEGG:00260; KEGG:00350; KEGG:00410; KEGG:00430; GO:0016830; GO:0009308; GO:0016831; GO:0005507	LOC100194731	—	Primary amine oxidase, liver isozyme	4.819376	2.68402
		cdo1	*ido2*	Cysteine dioxygenase type 1	4.322272	3.402714
		glyctk	*glyctk*	Glycerate kinase	3.421706	1.619655
		LOC106578921	*snca*	Alpha-synuclein	2.391534	1.492168
		gadl1	*gadl1*	Glutamate decarboxylase like 1	2.129529	1.394377
		LOC106590602	*hgd*	Homogentisate 1,2-dioxygenase	2.11657	1.271435
		LOC106594092	*gadl1*	Acidic amino acid decarboxylase GADL1	2.086634	1.401054
		LOC106577031	*ido2*	Indoleamine 2,3-dioxygenase 2	2.025267	1.813782
		LOC106601268	*aoc3*	Membrane primary amine oxidase	2.012616	1.86074
		LOC100194624	*aldoa*	Aldolase a, fructose-bisphosphate 1	1.380141	-1.93997
		LOC106569508	*gad1*	Glutamate decarboxylase 1	-1.59289	-1.52899
		dcup	*urod*	Uroporphyrinogen decarboxylase	-2.78764	-1.53059
Carbon dioxide transport	KEGG:00910; GO:0016835; GO:0006885; GO:0016836; GO:0004089; GO:0019755; GO:0015670; GO:0015106; GO:0015701	*ca6*	*ca6*	Carbonic anhydrase VI	3.912092	1.437486
		LOC106568872	—	Carbonic anhydrase-related protein	2.221919	1.292131
		LOC100380859	*slc4a1*	Band 3 anion exchange protein	-1.70173	-1.67966
		LOC106606856	*slc4a1*	Band 3 anion exchange protein	-1.77448	-1.74367
		LOC106600435	*aqpa*	Aquaporin FA-CHIP	-1.84931	-1.40969
		hem4	*uros*	Uroporphyrinogen-III synthase	-1.91692	-1.456
		slc39a4	*slc39a4*	Solute carrier family 39 member 4	-2.17046	-1.6271
		cahz	*ca*	Carbonic anhydrase	-2.37238	-1.65257
		LOC106590208	*ca1*	Carbonic anhydrase 1	-3.36955	-2.36895
Heme biosynthetic process	KEGG:00860; GO:0016765; GO:0046148; GO:0033013; GO:0042168; GO:0006778; GO:0033014; GO:0006783; GO:0006779; GO:0046501; GO:0006782	LOC106600953	*ftm*	Ferritin, middle subunit	1.483139	1.299958
		fech	*fech*	Ferrochelatase	-1.48545	-1.33441
		hem4	*uros*	Uroporphyrinogen-III synthase	-1.91692	-1.456
		alad	*alad*	Aminolevulinate dehydratase	-1.92556	-1.52312
		LOC106605668	*ppox*	Protoporphyrinogen oxidase	-2.2272	-1.76897
		LOC106590880	*mgst1*	Microsomal glutathione S-transferase 1	-2.22977	-1.47499
		hmbsa	*hmbsa*	Hydroxymethylbilane synthase a	-2.27196	-1.60444
		dcup	*urod*	Uroporphyrinogen decarboxylase	-2.78764	-1.53059
		LOC106575060	*mgst1*	Microsomal glutathione S-transferase 1	-3.11463	-1.83046
		LOC123729220	*hmbs*	Porphobilinogen deaminase	-3.11501	-1.75787

Plasma lipoprotein particle clearance	GO:0097006; GO:0034381; GO:1990777; GO:0034358; GO:0055102; GO:0034385; GO:0042157; GO:0004859; GO:0005504; GO:0015918; GO:0033293; GO:0030301	LOC106575194	—	Phospholipase A2 inhibitor 31 kDa subunit	6.383575	4.350139
		LOC106577511	*apoc1*	Apolipoprotein C-I	4.17644	3.648967
		apoeb	*apoeb*	Apolipoprotein Eb	3.658369	2.538333
		gramd1ba	*gramd1ba*	GRAM domain containing 1Ba	3.297985	2.531747
		ret7	*rbp7*	Retinoid-binding protein 7	2.807238	1.903703
		apoc1	*apoc1*	Apolipoprotein C-I	2.761315	2.155935
		sr-bi	*scarb1*	Scavenger receptor class B type I	1.5873	1.253306
		LOC106565680	*pigq*	Phosphatidylinositol glycan anchor biosynthesis, class Q	-1.64591	-1.3415
		LOC106593670	*pigq*	Phosphatidylinositol N-acetylglucosaminyltransferase subunit Q	-2.44308	-1.64622
Gas transport	GO:0098754; GO:0009636; GO:0016684; GO:0019825; GO:0031720; GO:0043177; GO:0046906; GO:0072562; GO:0072593; GO:0005833; GO:1990748; GO:0015669; GO:0020037; GO:0042743; GO:0097237; GO:0042744; GO:0098869; GO:0015671; GO:0016209; GO:0005344; GO:0004601	LOC106577511	*apoc1*	Apolipoprotein C-I	4.17644	3.648967
		ret7	*rbp7*	Retinoid-binding protein 7	2.807238	1.903703
		apoc1	*apoc1*	Apolipoprotein C-I	2.761315	2.155935
		LOC106577031	*ido2*	Indoleamine 2,3-dioxygenase 2	2.025267	1.813782
		LOC106564824	*cat*	Catalase	-1.26298	-1.30092
		LOC106594777	*hba4*	Hemoglobin subunit alpha-4	-1.60007	-1.63189
		LOC106586175	*cat*	Catalase	-1.61779	-1.57397
		LOC106600435	*aqpa*	Aquaporin FA-CHIP	-1.84931	-1.40969
		LOC106595268	*hbb*	Hemoglobin subunit beta	-1.85066	-1.61304
		LOC106592262	*cyp4f3*	Cytochrome P450 4F3	-1.98163	-1.32562
		LOC123724067	*hbb*	Hemoglobin subunit beta	-2.01385	-1.92167
		cahz	*ca*	Carbonic anhydrase	-2.37238	-1.65257
		LOC100136576	*hbb*	Beta globin	-2.38236	-1.45305
		LOC123723657	*hbb*	Hemoglobin subunit beta	-2.50303	-1.7005
		LOC106601072	*hba1*	Hemoglobin subunit alpha	-2.61667	-2.0076
		LOC106601051	*hbb*	Hemoglobin subunit beta	-2.69688	-1.32678
		LOC106607375	*hba4*	Hemoglobin subunit alpha-4	-2.71141	-1.37278
		LOC106607372	*hbb*	Hemoglobin subunit beta	-2.71142	-1.29564
		hba	*hba1*	Hemoglobin subunit alpha	-2.77086	-2.63751
		LOC106601071	*hba4*	Hemoglobin subunit alpha-4	-2.77092	-1.97134
		LOC106601074	*hbb1*	Hemoglobin subunit beta-1	-2.90168	-1.78408

	LOC106601077	*hba1*	Hemoglobin subunit alpha	-2.91508	-1.90858
	LOC106607236	*hbb1*	Hemoglobin subunit beta-1	-3.09796	-3.2593
	LOC106564853	*epx*	Eosinophil peroxidase	-3.10406	-1.38847
	LOC106607371	*hbb1*	Hemoglobin subunit beta-1	-3.15182	-1.53555
	LOC106607373	*hba1*	Hemoglobin subunit alpha	-3.32986	-1.51014
	LOC106590208	*ca1*	Carbonic anhydrase 1	-3.36955	-2.36895
	LOC106607380	*hbb1*	Hemoglobin subunit beta-1	-3.8194	-1.61304

Leading: L; sub-leading: SL

Supplementary Table 5. Summary of leading GO terms/KEGG pathways enriched based on shared DEGs related to pairwise comparisons between all families infested with adult or chalimus stages of lice at 20 °C versus 10 °C (T20A vs. T10A | T20C vs. T10C) in the skin.

**GO Description (L)**	**GO/KEGG ID (L + SL)**	**Gene ID**	**Gene symbol**	**Gene description**	**Fold-change (log_2FC)**	
					T20A vs. T10A (all families)	T20C vs. T10C (all families)
Collagen fibril organization	GO:0030199	LOC106581278	*serpinh1*	Serpin H1	4.157571	2.917116
		LOC106613072	*serpinh1*	Serpin H1	3.743492	2.463422
		LOC106599956	—	Hemagglutinin/amebocyte aggregation factor	2.421783	1.61961
		LOC106585899	*lox*	Protein-lysine 6-oxidase	-4.23847	-1.57433
Collagen catabolic process	GO:0030574	LOC106581219	*mmp13*	Collagenase 3	-1.30266	-1.62173
		mmp9	*mmp9*	Matrix metalloproteinase 9	-1.649	-2.56856
		LOC106566399	*mmp9*	Matrix metalloproteinase-9	-1.92883	-1.60336
Copper ion binding	GO:0005507	LOC106578921	*snca*	Alpha-synuclein	3.562443	1.747009
		LOC100194731	—	Primary amine oxidase, liver isozyme	3.396658	1.701452
		LOC106585899	*lox*	Protein-lysine 6-oxidase	-4.23847	-1.57433
Beta-Alanine metabolism	KEGG:00410	LOC100194731	—	Primary amine oxidase, liver isozyme	3.396658	1.701452
		LOC106594092	*gadl1*	Acidic amino acid Decarboxylase GADL1	2.956788	1.25299
		gadl1	*gadl1*	glutamate decarboxylase like 1	2.756033	1.5421
Response to heat	GO:0009266; GO:0009408; GO:0140662	LOC106608136	*hsp90aa1*	Heat shock protein HSP 90-alpha	3.485054	1.618535
		hs90a	*hsp90aa1*	Heat shock protein HSP 90-alpha	3.59319	1.518328
		dnaja	*dnaja*	DnaJ heat shock protein family (Hsp40) member A	2.751812	2.485168
		hsc70	*hspa8*	Heat shock cognate 70	1.730726	1.799879
Hemoglobin complex	GO:0098754; GO:0009636;	LOC106577511	*apoc1*	Apolipoprotein C-I	3.45601	2.440826
		LOC106611713	*cybb*	cytochrome b-245 heavy chain	1.691215	1.518675

GO:0016684; GO:0019825; GO:0031720; GO:0043177; GO:0072562; GO:0072593; GO:0005833; GO:1990748; GO:0015669; GO:0042743; GO:0097237; GO:0042744; GO:0098869; GO:0015671; GO:0016209; GO:0005344; GO:0004601	LOC106601077	*hba1*	Hemoglobin subunit alpha	-1.82997	-1.44173
	LOC106601072	*hba1*	Hemoglobin subunit alpha	-1.86314	-1.36565
	LOC106601074	*hbb1*	Hemoglobin subunit beta-1	-2.06566	-1.3783
	LOC106607372	*hbb*	Hemoglobin subunit beta	-2.13024	-1.28585
	LOC106607373	*hba1*	Hemoglobin subunit alpha	-2.29027	-1.54982
	apoc1	*apoc1*	Apolipoprotein C-I	-2.84485	-1.89876
	LOC106607371	*hbb1*	Hemoglobin subunit beta-1	-2.84691	-1.45005

Leading: L; sub-leading: SL
